# Rich polymorphism in nicotinamide revealed by melt crystallization and crystal structure prediction

**DOI:** 10.1038/s42004-020-00401-1

**Published:** 2020-11-04

**Authors:** Xizhen Li, Xiao Ou, Bingquan Wang, Haowei Rong, Bing Wang, Chao Chang, Baimei Shi, Lian Yu, Ming Lu

**Affiliations:** 1 School of Pharmaceutical Sciences, Sun Yat-sen University, Guangzhou, China; 2 Shenzhen Jingtai Technology Co., Ltd. (XtalPi Inc.), Shenzhen, China; 3 School of Pharmacy, University of Wisconsin – Madison, Madison, WI USA; 4 Guangdong Provincial Key Laboratory of New Drug Design and Evaluation, Sun Yat-sen University, Guangzhou, China

**Keywords:** Crystal engineering, Structure prediction, Computational chemistry

## Abstract

Overprediction is a major limitation of current crystal structure prediction (CSP) methods. It is difficult to determine whether computer-predicted polymorphic structures are artefacts of the calculation model or are polymorphs that have not yet been found. Here, we reported the well-known vitamin nicotinamide (NIC) to be a highly polymorphic compound with nine solved single-crystal structures determined by performing melt crystallization. A CSP calculation successfully identifies all six *Z*′ = 1 and 2 experimental structures, five of which defy 66 years of attempts at being explored using solution crystallization. Our study demonstrates that when combined with our strategy for cultivating single crystals from melt microdroplets, melt crystallization has turned out to be an efficient tool for exploring polymorphic landscapes to better understand polymorphic crystallization and to more effectively test the accuracy of theoretical predictions, especially in regions inaccessible by solution crystallization.

## Abstract (web summary)

Nicotinamide is commonly used to form cocrystals with drugs, but its crystal polymorphism is poorly understood. Here seven polymorphs, along with two known forms, are identified by melt crystallisation.

## Introduction

Polymorphism has attracted increasing attention from both academic researchers and those in industry^1–3^. Recently, significant progress has been made in the experimental discovery and theoretical prediction of crystal polymorphs^3–8^. Despite these advances, a key issue in this area is that computational predictions usually yield far more possible polymorphs than are known, raising the famous question posed by Price, “Why don’t we find more polymorphs?”^9^. It is unclear whether the answer to Price’s question lies in theory (insufficient removal of implausible structures), in experiments (failure to observe certain polymorphs because of slow nucleation, slow growth, instability, or other factors), or in both.

Melt crystallization is known to reveal several polymorphs that are not accessible by solution crystallization^10–16^. We recently reported a general method to cultivate single crystals from a supercooled melt, thus helping to solve single-crystal structures of melt-crystallized polymorphs^17^. This progress will facilitate the elucidation of additional polymorphic structures of organic compounds that escape solution crystallization.

**Fig. 1:**
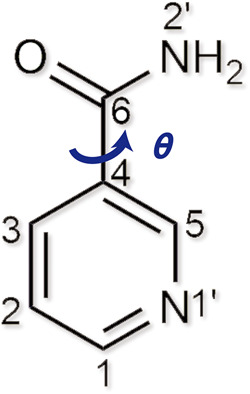
Molecular structure of NIC. The conformation difference between nicotinamide polymorphs is most pronounced in the torsion angle *θ*(C5-C4-C6-N2′).

Nicotinamide (NIC) is a naturally occurring form of vitamin B3 (Fig. 1). In recent decades, NIC has become an extensively used coformer for forming cocrystals with drugs to modify their solubility or other properties. A search in the Cambridge Structural Database (CSD) (version 5.41 updated (May 2020)) returned more than 100 structures of organic cocrystals with NIC as a conformer. Although the study of NIC polymorphism began in 1943^18,19^ and thousands of NIC-related cocrystal screening experiments have been performed by various laboratories, only two polymorphic structures of NIC have been reported: the first crystal structure (here denoted by α) in 1954^20^, and the second structure (here denoted by β) in 2011^21^. The latter was found during an attempt to co-crystallize NIC with the antitubercular drug isoxyl.

Here, we report seven polymorphic structures of this well-known vitamin, in addition to two known structures, by performing melt crystallization. Single crystals of the nine polymorphs were grown from melt microdroplets, and all crystal structures were successfully determined by single-crystal X-ray diffraction (SCXRD). All six experimental structures with one or two molecules in the asymmetric unit (*Z*′ = 1 and 2) were found in the lattice energy landscape calculated by crystal structure prediction (CSP). These findings indicate the potential for melt crystallization to find hidden polymorphs and the prospect of a convergence between experiment and theory.

## Results

### Discovery of NIC polymorphism

**Fig. 2:**
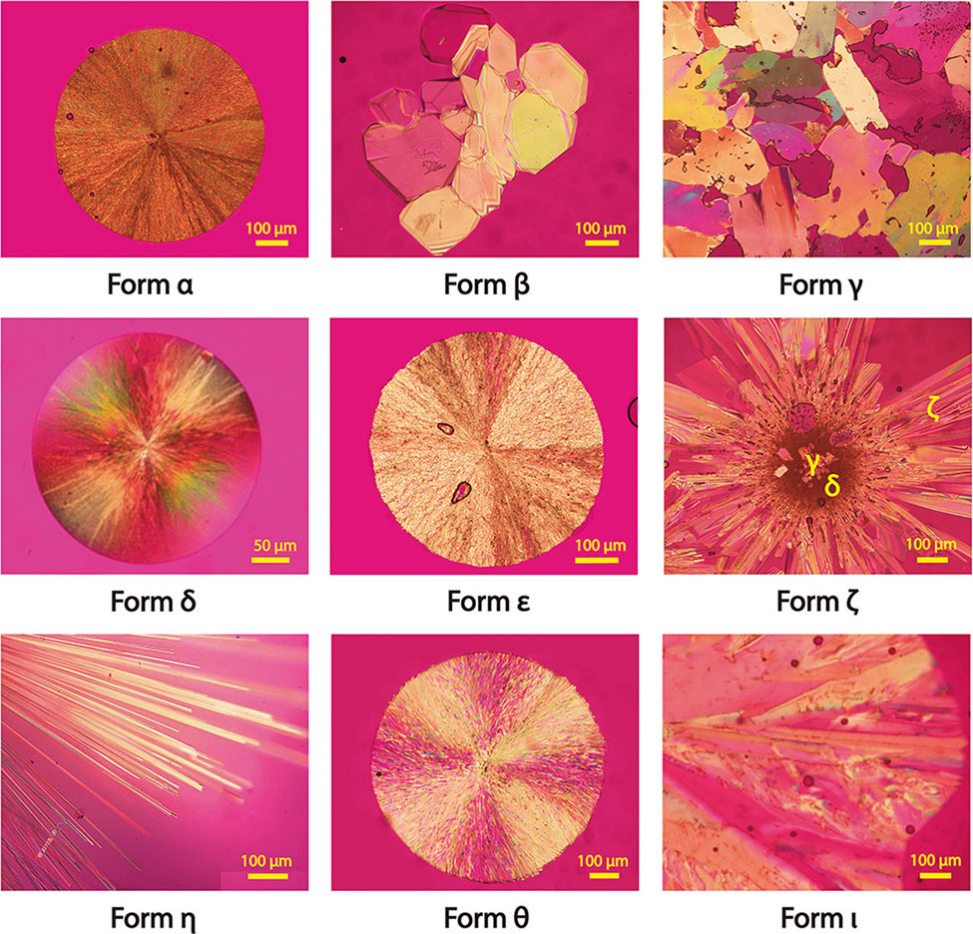
Polarized optical microscopy images of NIC polymorphs. All nine polymorphs were observed by melt crystallization.

A comprehensive polymorph search was conducted by melt crystallization. Surprisingly, nine NIC polymorphs were obtained from the supercooled melt, including seven new forms (γ, δ, ε, ζ, η, θ, and ι) and the previously known forms (α and β). These polymorphs were identified by morphologies, melting points, powder X-ray diffraction (PXRD) patterns, Raman and Fourier transform infrared (FTIR) spectra (Fig. 2 and Supplementary Figs. 1–3).

**Fig. 3:**
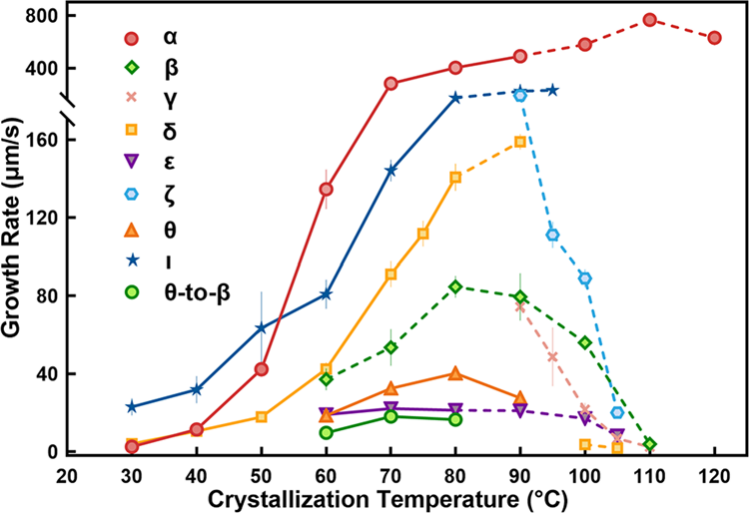
Crystal growth rates of NIC polymorphs as a function of temperature. The solid lines indicate that these polymorphs could spontaneously nucleate from the free melt at the corresponding temperature regions. Otherwise, the data points are connected by dashed lines. Red circles: form α; green diamonds: form β; pink cross marks: form γ; yellow squares: form δ; purple inverted triangles: Form ε; light blue hexagons: form ζ; orange triangles: form θ; blue stars: form ι; green circles: θ-to-β solid-solid conversion. The growth rates of form δ at 100 °C were measured using a melt microdroplet sample, while others were measured using samples sandwiched between two coverslips. The crystallization experiment at each temperature was repeated at least three times.

Form α is the most stable polymorph. It crystallized with a very high growth rate (768 ± 48 μm/s at 110 °C) (Fig. 3). Once this form nucleated, it rapidly consumed the remaining liquid and triggered the phase transition of any metastable polymorphs into Form α. Of the eight kinetic forms, only four (δ, ε, η, and θ) were usually observed to self-nucleate from the NIC melt in certain temperature regions, which are marked with solid lines in Fig. 3. Below 60 °C, form δ was the dominant polymorph. At 60–80 °C, forms δ, ε and θ often concomitantly nucleate from the NIC melt. Form ε crystallized with the lowest growth rate. Forms δ and θ were unstable and usually converted to the more stable forms γ and β, respectively. Above 90 °C, it was difficult for most polymorphs to spontaneously nucleate, except for form η, which was observed to crystallize at 104 °C with an extremely low nucleation probability. Form η usually crystallized as needle crystals (Fig. 2) because the growth temperature (104 °C) is very close to its melting point (108 °C, determined by polarized optical microscopy (POM)).

**Fig. 4:**
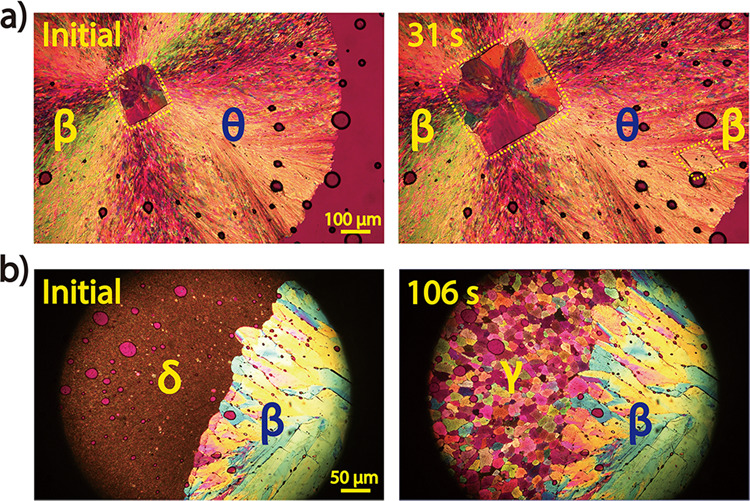
Formation of Forms β and γ via solid-solid phase conversion. **a** NIC melt crystallized first as form θ and then transformed to form β at 70 °C. **b** Form δ was extremely unstable and rapidly converted to form γ at 75 °C.

Forms β and γ were not observed to self-nucleate from the NIC melt but only crystallized as a result of polymorphic transformation from less stable polymorphs, usually forms θ and δ, respectively (Fig. 4). Both δ-to-γ and θ-to-β solid-solid phase conversions occurred spontaneously, but the nucleation densities of the daughter polymorphs were quite different. Form γ nucleated very densely from form δ and grew as crowded colourful granules (Fig. 4b), while form β nucleated from form θ very sparsely and grew with a regular shape (Fig. 4a). The induction time of θ-to-β conversion increased as the temperature increased (7 ± 3 s at 60 °C, 25 ± 11 s at 70 °C, and 58 ± 21 s at 80 °C, *n* = 15). Forms β and γ were determined as the second and third most stable polymorphs, respectively, according to the melting points (Table 1) and the experimentally observed polymorphic conversions. Therefore, these two polymorphs of NIC were suggested to be thermodynamically favoured but kinetically hindered polymorphs, similar to Form YT04 of 5-methyl-2-[(2-nitrophyenyl)amino]-3-thiophenecarbonitrile (ROY)^22^.

**Fig. 5:**
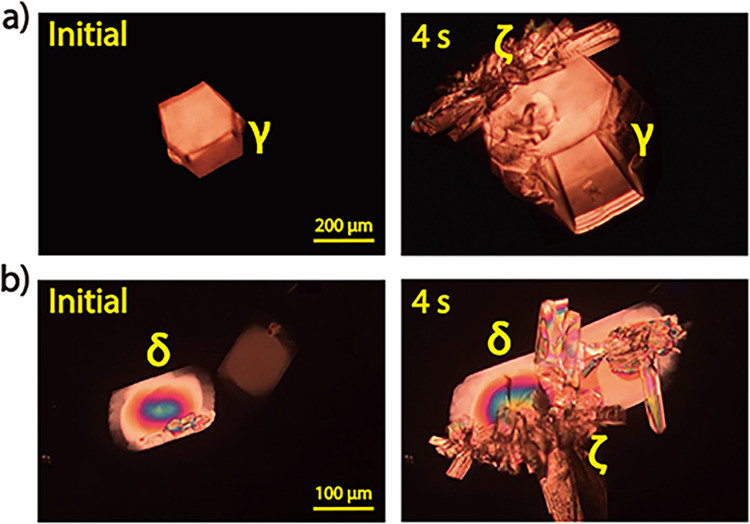
Formation of form ζ via cross-nucleation. Single crystals of forms γ (**a**) and δ (**b**) were grown first at 114.5 and 114 °C, respectively, and then transferred to 94 and 100 °C, respectively, to trigger the cross-nucleation of form ζ.

In addition to forms β and γ, form ζ is the third polymorph that cannot self-nucleate from the NIC melt. This polymorph usually cross-nucleated on the surface of form γ at 90–95 °C (Fig. 5a) or on that of form δ at 90–105 °C (Fig. 5b). Interestingly, forms γ, δ and ζ could cross-nucleate at different temperatures. As forms γ and ζ were transferred to 50–80 °C, they triggered the nucleation of form δ (Supplementary Fig. 4). At 90–95 °C, forms δ and ζ interactively cross-nucleated on each other’s surface and thus grew together (Supplementary Fig. 5). Most of the complicated cross-nucleation phenomena occurring in the NIC system can be explained by the different growth rates (shown in Fig. 3) and the known rule that cross-nucleation is motivated by kinetic benefit, where the new polymorph always grows faster than or as fast as the initial polymorph^23^.

**Fig. 6:**
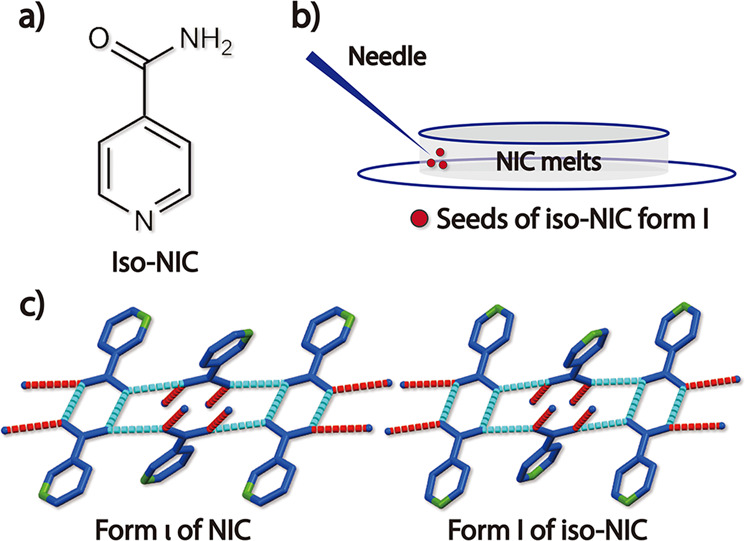
Discovery of form ι via pseudoseeding. **a** Molecular structure of iso-NIC. **b** Acquisition of NIC form ι by seeding iso-NIC form I in the NIC melt at 70–95 °C. **c** Hydrogen bonds in NIC form ι and iso-NIC form I.

Inspired by the success of pseudoseeding^24–26^, isonicotinamide (iso-NIC), a compound structurally similar to NIC (Fig. 6a), was selected as a potential template molecule. The commercially available powder of iso-NIC (form I) was seeded at the edge of the NIC melt that was sandwiched between two coverslips at 70–95 °C. This successfully triggered the crystallization of another new polymorph (the ninth) of NIC, designated form ι (Supplementary Fig. 6). Form ι had the lowest melting point among the nine NIC polymorphs and usually quickly transformed to form α (Supplementary Fig. 6). This ι-to-α conversion typically sped up as the temperature decreased to 70 °C. Form ι rarely self-nucleated from the NIC melt. We observed the spontaneous nucleation of form ι in only one sample after hundreds of experiments. By repeatedly melting this sample and then crystallizing it at different temperatures, we measured the growth rates of form ι in the temperature range of 30–80 °C (Fig. 3).

### Single-crystal structure solution of NIC polymorphs

**Fig. 7:**
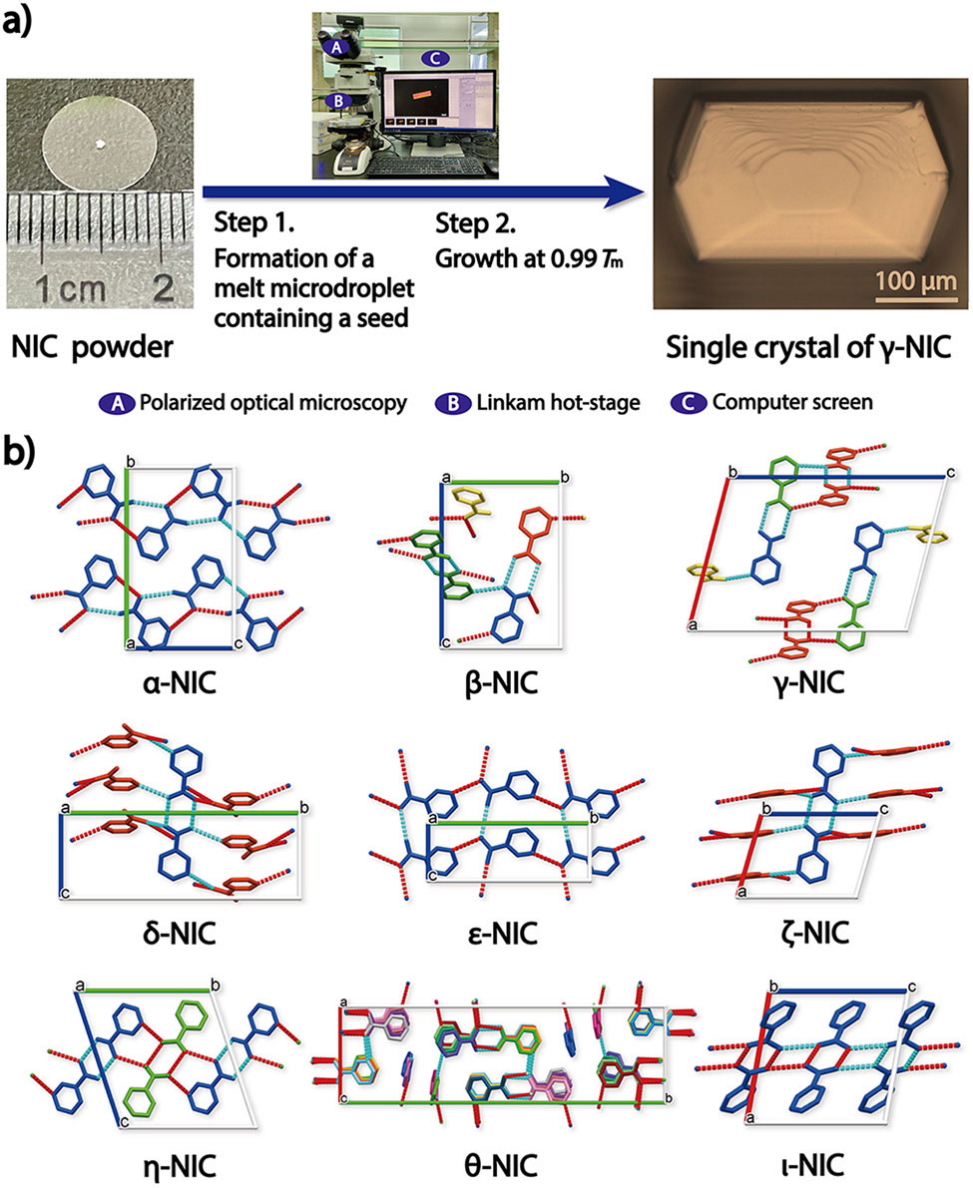
Single-crystal structure determination of NIC polymorphs. **a** Cultivation of a single crystal of γ-NIC using hot-stage microscopy. **b** Crystal structures of nine NIC polymorphs determined by SCXRD at 100 K using single crystals grown from melt microdroplets. Forms α and β were redetermined for comparison. Colours are used to differentiate crystallographically independent molecules. Forms α, β, δ, ε and η are shown along the *a*-axis; Forms γ, ζ, and ι are shown along the *b*-axis; Form θ is shown along the *c*-axis.

Since melt crystallization generally produces crystalline spherulites of insufficient quality for SCXRD investigations, we developed a microdroplet strategy for cultivating single crystals from melt^17^. Following the partial melting of NIC polycrystals, a single-crystal seed was allowed to grow in an isolated microdroplet at 0.98–0.99 *T*_m_ (here, *T*_m_ refers to the melting point) to avoid the occurrence of noncrystallographic branching and interference from other crystals. Using this method, we successfully grew single crystals of all the NIC polymorphs to a sufficient size and quality for acquiring good SCXRD data (for POM images and cultivation conditions see Supplementary Fig. 7 and Supplementary Table 1, respectively) and thus determined their single-crystal structures (Fig. 7 and Table 1). The PXRD patterns simulated from the solved single-crystal structures were compared with the experimental PXRD data (Supplementary Fig. 8). The results confirmed that crystal structures of NIC polymorphs were correctly determined. The slight differences between the simulated data and experimental data might be induced by the high orientation of crystal growth in melt crystallization, the possible impurity of the phase, and the temperature difference between the SCXRD experiment (100 K) and the PXRD experiment (298 K).

**Table 1:** Discovery and structures of NIC polymorphs.

Polymorphs	Form α	Form β	Form γ	Form δ	Form ε	Form ζ	Form η	Form θ	Form ι
Discov. method^a^ [ref]	SC^20^	SC^21^	MC/PT	MC	MC	MC/CN	MC	MC	MC/CN
yr. str. Solved,	1954	2011	This work	This work	This work	This work	This work	This work	This work
Method^b^ [ref]	SCXRD^20^	SCXRD^21^	SCXRD	SCXRD	SCXRD	SCXRD	SCXRD	SCXRD	SCXRD
NICOAM No.^c^	1st str.	04	NA	NA	07	NA	08	09	NA
CCDC number^d^	1,866,090	1,866,091	1,866,092	1,984,661	1,893,048	1,984,570	1,893,049	1,893,061	1,984,664
*T*, K	100	100	100	100	100	100	100	100	100
Crystal system	Monoclinic	Monoclinic	Monoclinic	Monoclinic	Monoclinic	Triclinic	Triclinic	Monoclinic	Monoclinic
Space group	*P*2_1_/c (14)	*P*2/n (13)	*P*2_1_/c (14)	*P*2_1_/c (14)	*P*2_1_ (4)	*P*$\bar 1$ (2)	*P*$\bar 1$ (2)	*P*2_1_ (4)	*P* 2_1_/c (14)
a (Å)	3.882666(15)	14.9915 (3)	15.3671(7)	7.3525(3)	3.81231(4)	7.5564(2)	3.7525(3)	10.70050(10)	9.9011(3)
b (Å)	15.6453(5)	10.6814 (2)	7.3847(5)	20.7383(9)	14.38794(19)	7.9413(2)	12.3229(7)	35.1874(2)	5.87732(19)
c (Å)	9.3836(3)	15.1888 (4)	21.1950(10)	7.4058(4)	5.11942(6)	10.7974(4)	13.0618(6)	15.82560(10)	10.2784(5)
α (deg)	90	90	90	90	90	108.100(3)	71.499(5)	90	90
β (deg)	98.394(4)	101.955(2)	104.650(5)	91.044	94.2560(10)	102.596(2)	85.676(6)	102.5800(10)	100.003
γ (deg)	90	90	90	90	90	98.293(2)	85.202(5)	90	90
Volume (Å^3^)	563.904(4)	2379.43(9)	2327.0(2)	1129.04	280.032	585.137	570.013	5813.37	589.028
*Z*	4	16	16	8	2	4	4	40	4
*Z*’	1	4	4	2	1	2	2	20	1
*ρ* (g cm^−3^)	1.439	1.364	1.394	1.437	1.448	1.386	1.423	1.395	1.377
R-Factor (%)	5.38	3.65	7. 49	6.09	2.51	5.45	6.12	6.04	6.40
*T*_m_ (deg)^e^	129	116.5	115	114	110.5	109.5	108	104.5	103
Nucleation in MC^[f]^	SN	PT	PT	SN	SN	CN	SN	SN	CN
CSP rank	1	Not searched	Not searched	7	8	92	6	Not searched	11
RMSD_15_^g^ (Å)	0.228	0.077	0.045	0.105	0.107	0.811	0.135	*0.038*	0.202

^a^Methods of polymorph discovery: *SC* solution crystallization, *MC* melt crystallization, *PT* polymorphic transformation, *CN* cross-nucleation.

^b^Methods of structural solution: *SCXRD* single-crystal X-ray diffraction.

^c^NICOAM is the root name for nicotinamide polymorphs in the CSD; *NA* NICOAM number is not available at present.

^d^The CCDC numbers and the crystallographic parameters of α-NIC and β-NIC presented in Table 1 are of the single-crystal structures redetermined in this work.

^e^Melting points of nine polymorphs were determined using hot-stage microscopy. The error in *T*_m_ is 1 °C based on at least three measurements.

^f^Nucleation mode in melt crystallization: *SN* self-nucleation, *PT* polymorphic transformation, *CN* cross-nucleation.

^g^For forms α, δ, ε, ζ, η, and ι, RMSD_15_ is calculated between the experimental structure and the predicted structure using Mercury software; for forms β, γ and θ, RMSD_15_ is calculated between the experimental structure and the optimized structure, as these polymorphs are not included in the CSP search.

NIC has two hydrogen bond acceptors (the amide oxygen and pyridine nitrogen) and two donors (the amide hydrogens), almost all of which are involved in the formation of hydrogen bonds in every polymorph except for form ι. Hydrogen bonding results in amide dimers in all of the polymorphs except for forms α and ε. Form ι shows extremely similar crystal packing and hydrogen bonding to that of its parent phase, iso-NIC form I (Fig. 6c). This structural similarity can be fully explained by the fact that the only difference in the chemical structures of the two compounds is the position of the pyridine nitrogen, and this nitrogen atom is not involved in the formation of hydrogen bonds in either structure. Our study also revealed a rare *Z’* = 20 structure formed in form θ. In addition to θ-NIC, there are only three other organic compounds having *Z’* ≥ 20 structures in the CSD (version 5.41, updated May 2020); Refcodes VUJBAE (*Z’* = 20), OFEREZ (*Z’* = 24), and OGUROZ (*Z’* = 56).

**Fig. 8:**
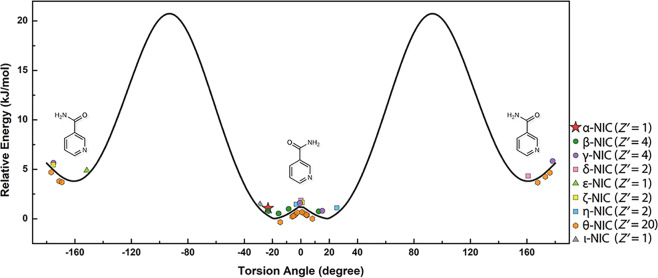
Conformational energy as a function of the torsion angle *θ* (C5-C4-C6-N2′) obtained from the relaxed scan. The calculation was conducted at the B3LYP/6-31G(d) level^46,47^ using the program GAUSSIAN 09^48^. These calculation results agree well with previously reported data^50–52^. The experimental conformations found in the nine determined NIC polymorphs are plotted along the two sides of the curve using different colours and symbols. Form θ has twenty crystallographically independent molecules, and thus, it is difficult to avoid the overlay of its data points.

The nine polymorphs of NIC have many different conformations with different torsion angles *θ* (C5-C4-C6-N2′) (Supplementary Table 2). All these crystallographic conformations are related to two gas-phase conformers: the global minimum-energy confrmer ((*E*)-Form) with *θ* ≈ ± 18° (0 kJ/mol) and the local minimum-energy conformer ((*Z*)-Form)) with *θ* ≈ 180 ± 20° (3.6 kJ/mol) (Fig. 8). Therefore, forms α/β/η/ι were determined to be related to Forms γ/δ/ε/ζ/θ by conformational polymorphism^27^. In summary, a variety of conformations, hydrogen bonding modes and crystal packings (Supplementary Fig. 9) were found to contribute to the rich polymorphism of NIC. This system is a clear contrast with ROY, as NIC is a molecule with a plurality of hydrogen bonding motifs and less conformational variability.

### Crystal structure prediction of NIC

**Fig. 9:**
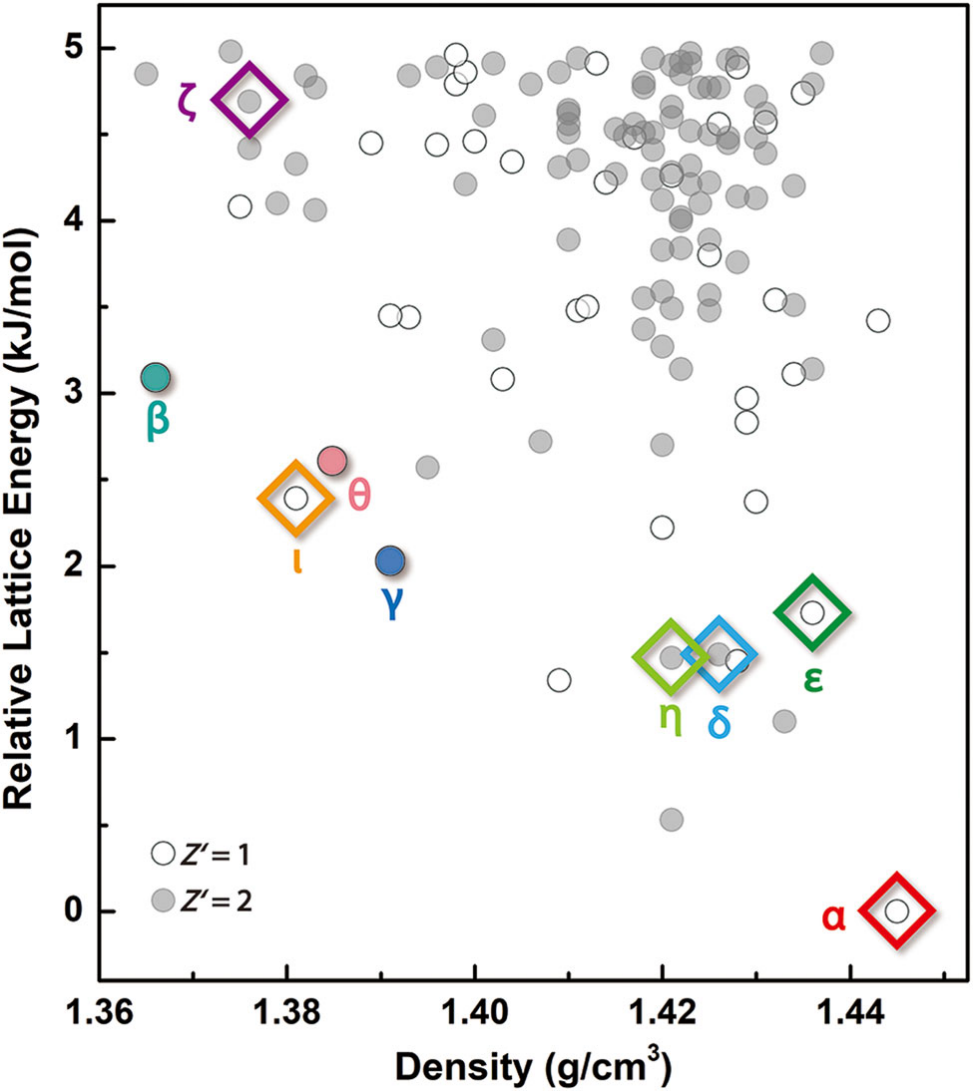
Crystal energy landscape of NIC with *Z*’ = 1 (○) and *Z*’ = 2 (●). All six *Z*’ = 1 and 2 experimental structures were found and are marked with diamond-shaped symbols and the names of the corresponding polymorphs. Two *Z*’ = 4 structures (Forms β and γ) and one *Z*’ = 20 structure (Form θ) are also marked with dots in this landscape after being optimized and evaluated using the optPBE-vdw functional.

A CSP calculation of NIC was performed using an in-house cloud platform^28^. Considering the computational cost, CSPs with *Z*’ = 1 and 2 were performed over the space groups that are most frequently observed in achiral organic crystals (11 space groups for *Z*’ = 1 and 6 space groups for *Z*’ = 2). Crystal packing possibilities were surveyed for a range of NIC conformations that were primarily defined by the amide-pyridine torsion angle. A system-specific force field was created and applied in the initial generation of possible low-energy structures, and this was followed by further optimization and evaluation using the Perdew–Burke–Ernzerh functional^29^ with the dispersion correction (optPBE-vdw)^30^ implemented in VASP software^31–33^. Finally, 124 thermodynamically plausible structures within +5 kJ/mol of the global minimum were obtained and are shown in Fig. 9 and Supplementary Table 3.

The predicted structures were compared with the experimental results. This comparison identified all three *Z*’ = 1 (α, ε, ι) and all three *Z*’ = 2 (δ, ζ, η) NIC polymorphs that were observed in our experiments (Fig. 9). The geometry of the predicted structure reasonably agreed with the experimental structure of Form ζ, with a root mean square distance between the overlays of 15 molecules (RMSD_15_) of 0.811 Å; in all other cases, the agreement was excellent (RMSD_15_ < 0.3 Å) (Supplementary Table 4). Form α was identified as the global minimum with an RMSD_15_ = 0.228 Å. However, the predicted lattice energy ranking at 0 K (α > η > δ > ε > γ > ι > θ > β > ζ) differed greatly from the experimental ranking obtained based on the heat of fusion (α > β (by 2.5 kJ/mol) > γ (by 3.0 kJ/mol) > ε (by 3.6 kJ/mol), Supplementary Fig. 10 and Supplementary Table 5) or the order of polymorphic transformation (α > β > γ > ε > η > θ, Supplementary Figs 11–19). Such a discrepancy can arise from the neglect of temperature and the precision of the energy calculation, as in this case. Nevertheless, the accurate identification of the low-energy crystal structures corresponding to all six experimental polymorphs for the class considered (i.e., *Z*′ = 1 and 2) highlights the significant methodological advances that have been achieved in recent years^6–8,34–36^. If this CSP calculation had been performed before this experimental work, these five structures would have appeared to be the results of overprediction, whereas in reality, they would have been present but unable to be experimentally observed at that time. This NIC case emphasizes the importance of performing experiments at discovery on hidden polymorphs to more effectively test the accuracy of theoretical models. Because NIC has two *Z*’ = 4 and one *Z*’ = 20 structures at this time, this molecule is suitable to be used as a model compound for testing the capability of the theoretical model to predict high *Z*’ structures. CSP-generated structures with no experimental analogue motivate further efforts on both the computational and experimental sides. Because additional polymorphs of ROY have been discovered^4,5,37^ or structurally characterized^38^ since the publication of Vasileiadis’s CSP^39^ and have matched the prediction results well, it is reasonable to expect that additional predicted NIC structures might also be observed experimentally with the efforts of more research groups. Given that nine polymorphs were discovered by melt crystallization at ambient pressure, pressure could be an additional dimension to explore^3,40,41^.

## Discussion

Why does melt crystallization produce many more polymorphs of NIC than solution crystallization? In general, the high molecular concentration in a melt results in a much higher thermodynamic driving force for nucleation than that in solution, facilitating the nucleation of metastable polymorphs. These transient forms can transform into more stable crystal forms and/or cross-nucleate a fast-growing phase, revealing more polymorphs, for example, the formation of forms β, γ, and ζ of NIC and the discovery of polymorphs R05^23^ and YT04^22^ of ROY. These metastable phases can be directly observed and then isolated for further structural characterization before phase conversion occurs. All of these factors facilitate the discovery of kinetic polymorphs that are difficult to obtain from solution. Recent work has revealed a growing list of polymorphs discovered by melt crystallization: paracetamol (3 of 9)^42^, ROY (4 of 13)^22,23,37^, griseofulvin (2 of 3)^43^ and vemurafenib (3 of 4)^12^. For NIC, 7 of 9 polymorphs were discovered by melt crystallization. The ability to grow single crystals from melt microdroplets further empowers the melt method, especially for exploring the high-energy region of the crystal energy landscape that is inaccessible using solution crystallization. This advance is important for establishing structure–property relationships using a diverse set of structures and for understanding the discrepancy between theoretically predicted and experimentally observed crystal structures.

In summary, we have presented our discovery of a nonamorphic system of NIC with nine solved single-crystal structures determined using melt crystallization. NIC has thus become the second most highly polymorphic compound that was structurally characterized by SCXRD. Compared with the record holder in this regard (ROY^4,5,38,44^ with 11 solved single-crystal structures) and three other compounds, aripiprazole (9)^26^, flufenamic acid (8)^45^ and galunisertib (5)^3^, NIC is a unique polymorphic system in that the discovery and single-crystal structure determination of seven new polymorphic structures were achieved through melt crystallization. We also provided a new example of a computer-predicted structure, ι-NIC, which was found by seeding a templating crystal, iso-NIC form I. Together with the success in predicting all the observed *Z*’ = 1 and 2 structures of NIC, this work indicates the prospect for improved agreement between theory and experiment. This motivates further progress in both areas to answer the famously posed question, “Why don’t we find more polymorphs?”.

## Methods

### Polymorph discovery and characterization

NIC and iso-NIC were purchased from Aladdin (Shanghai, China). Commercial NIC powders were placed between two coverslips and completely melted at 135 °C using a KER hot stage (3100–08S, China). Here, when we used the melt crystallization method, the melted sample was directly transferred from the KER hot stage to a Linkam hot stage (THM S600, Waterfield, UK) and isothermally crystallized at a preset temperature. When we used the cold crystallization strategy, the melted sample was first quenched on the surface of a metal block at room temperature and then transferred to a Linkam hot stage for crystallization. The diameters of the upper and lower coverslips were 6 mm and 10 mm, respectively. The purpose of this design was to avoid contacting NIC samples when moving the samples using tweezers, because the force of such a contact might trigger the nucleation of form α. A Nikon polarized optical microscope (eclipse lv100N pol, Tokyo, Japan) combined with the Linkam hot stage was employed to observe the crystallization behaviour and measure the crystal growth rate of each polymorph. The PXRD patterns of the NIC polymorphs were collected using a Bruker diffractometer (D8 Advanced, Madison, WI, USA) with Cu Kα radiation (*λ* = 1.5418 Å) at 40 kV and 40 mA. The scans were performed from 4.5° or 5° to 40° (2*θ*) at a rate of 6°/min. The as-received NIC sample was used to obtain the PXRD pattern of the α form. PXRD patterns of forms β, γ, δ, ε, ζ, η, and θ were obtained by using crystallographically pure samples. The preparation of crystallographically pure polymorphs is described in Supplementary Methods.

Raman spectra were collected using a Renishaw confocal Raman microscope (InVia Reflex, Renishaw plc, Gloucestershire, UK) with a 20× objective and a 514 nm excitation wavelength to give a laser spot diameter of approximately 20 μm. The exposure time was 10 ms, and the laser power was 100 or 50%. Spectra were collected and analysed using Renishaw WIRE 3.4 software. The wavelength scale was calibrated using silicon. The FTIR spectra of NIC polymorphs were collected in attenuated total reflectance (ATR) mode using a PerkinElmer FTIR spectrometer (Spectrum Two, Waltham, USA) with a PerkinElmer horizontal ATR accessory. The crystallographically pure samples were first prepared. After removing the upper coverslip, the sample was immediately placed on the ATR crystal and scanned at a resolution of 4 cm^−1^ from 400 to 3500 cm^−1^. Differential scanning calorimetry (DSC) thermograms were recorded using a NETZSCH DSC (200 F3 Maia, Selb, Germany) under a nitrogen atmosphere (40 mL/min). Samples were heated at 10 °C/min under a nitrogen purge. The data were analysed using Proteus software.

### Single crystal cultivation

Commercial NIC powders were placed on a coverslip to first prepare the crystallographically pure polycrystalline polymorph, or the desired polymorph with some low-melting-point crystal forms. This sample was partially melted around the melting point of the desired polymorph to form microdroplets each with a single-crystal seed in it, followed by cooling of this partial melt to let this seed grow into a single crystal with sufficient size and quality for SCXRD measurements (for details see Supplementary Table 1). The diameters of the microdroplets were controlled to be smaller than 1 mm.

### Single-crystal structure determination

Three-dimensional X-ray diffraction data of forms α, β and γ were collected using a Rigaku diffractometer (Oxford Diffraction Xcalibur Nova, Wroclaw, Poland), while diffraction data of the other six forms were collected using a Rigaku diffractometer (XtaLAB Synergy, Wroclaw, Poland). All data collections were performed at 100 K with Cu Kα radiation (*λ* = 1.54184 Å). Each single-crystal structure was solved by applying intrinsic phasing methods using SHELXT (Sheldrick, 2014) and subjected to full-matrix least-squares refinement using SHELXL (Sheldrick, 2016) with Olex 2. Torsion angles (*θ* (C5-C4-C6-N2)) of the nine NIC polymorphs (37 conformers) were calculated using Mercury 4.2.0. The simulated PXRD patterns were calculated using Mercury 4.2.0.

### Conformational energy scan

The conformational energy scan for torsion angle *θ* (C5-C4-C6-N2) was conducted at the B3LYP/6-31 G(d) level^46,47^ using the Gaussian 09 package^48^. The molecule was fully optimized with a fixed torsion angle for each step of 10°. Grimme’s empirical dispersion correction (known as Grimme-D3)^49^ was applied in the calculation.

### Crystal structure prediction

The crystal structure prediction for NIC was performed with *Z*′ = 1 for the 11 space groups, 1, 2, 4, 5, 9, 14, 15, 19, 29, 33, and 61 and *Z*′ = 2 for the 6 space groups, 2, 4, 14, 15, 19, and 61 in the cloud platform of XtalPi Inc., which integrates conformation analysis, force-field parameterization, crystal structure searching, clustering, and ranking^28^. The search space included lattice parameters, molecular positions and orientations, and rotations along some single bonds. Initially, a system-specific force field was created and applied in the initial generation of possible crystal structures, followed by further optimization and evaluation using the Perdew–Burke–Ernzerhof functional^29^ with dispersion correction (optPBE-vdw)^30^ implemented in VASP software^31–33^. The experimental structures of NIC determined by SCXRD were compared with 124 predicted structures with a cut-off of 5 kJ/mol using Mercury 4.2.0. An RMSD_15_ < 1.0 was used as the criterion for equality.

## Supplementary information

Supplementary Information

Description of Additional Supplementary Files

Supplementary Movie 1

Supplementary Data 1

Supplementary Data 2

## Data Availability

The data supporting the findings of this study are included in this article and its Supplementary Information. The single-crystal diffraction data of NIC polymorphs have all been deposited in the Cambridge Crystallographic Data Centre (CCDC) under deposition numbers CCDC 1866090 (Form α), CCDC 1866091 (Form β), CCDC 1866092 (Form γ), CCDC 1984661 (Form δ), CCDC 1893048 (Form ε), CCDC 1984570 (Form ζ), CCDC 1893049 (Form η), CCDC 1893061 (Form θ), and CCDC 1984664 (Form ι), respectively, and the relevant CIFs are provided as a file entitled “Supplementary Data 1”. These data can be obtained free of charge from The Cambridge Crystallographic Data Centre via www.ccdc.cam.ac.uk/data_request/cif. CSP-predicted structures are provided as a file named “Supplementary Data 2”. A video of the single-crystal growth of nicotinamide Form γ from a melt microdroplet is provided as a file entitled “Supplementary Movie 1”.

## References

[1] Cruz-Cabeza AJ, Reutzel-Edens SM, Bernstein J (2015). Facts and fictions about polymorphism. Chem. Soc. Rev..

[2] Rahal AO (2019). Polymorphism of ι-Tryptophan. Angew. Chem. Int. Ed..

[3] Bhardwaj RM (2019). A prolific solvate former, galunisertib, under the pressure of crystal structure prediction, produces ten diverse polymorphs. J. Am. Chem. Soc..

[4] Tyler AR (2020). Encapsulated nanodroplet crystallization of organic-soluble small molecules. Chem.

[5] Lévesque A, Maris T, Wuest JD (2020). ROY reclaims its crown: new ways to increase polymorphic diversity. J. Am. Chem. Soc..

[6] Greenwell C, Beran GJO (2020). Inaccurate conformational energies still hinder crystal structure prediction in flexible organic molecules. Cryst. Growth Des..

[7] Greenwell C (2020). Overcoming the difficulties of predicting conformational polymorph energetics in molecular crystals via correlated wavefunction methods. Chem. Sci..

[8] Hoja J (2019). Reliable and practical computational description of molecular crystal polymorphs. Sci. Adv..

[9] Price SL (2013). Why don’t we find more polymorphs?. Acta Crystallogr. Sect. B.

[10] Zhu Q (2016). Resorcinol crystallization from the melt: a new ambient phase and new “Riddles”. J. Am. Chem. Soc..

[11] Zhang S, Lee TWY, Chow AHL (2016). Crystallization of itraconazole polymorphs from melt. Cryst. Growth Des..

[12] Lu M, Taylor LS (2016). Vemurafenib: a tetramorphic system displaying concomitant crystallization from the supercooled liquid. Cryst. Growth Des..

[13] Shtukenberg AG (2017). Powder diffraction and crystal structure prediction identify four new coumarin polymorphs. Chem. Sci..

[14] Ciciliati MA, Eusébio MES, Silva MR, Cavalheiro ÉTG, Castro RAE (2019). Metoprolol: solid forms of a top selling antihypertensive. CrystEngComm.

[15] Shtukenberg AG (2019). Melt crystallization for paracetamol polymorphism. Cryst. Growth Des..

[16] Zhang K, Fellah N, Shtukenberg AG, Hu C, Ward MD (2020). Discovery of new polymorphs of the tuberculosis drug isoniazid. CrystEngComm.

[17] Ou X, Li XL, Rong HW, Yu L, Lu M (2020). A general method for cultivating single crystals of small organic compounds from melt microdroplets. Chem. Commun..

[18] Kofler L, Kofler A (1943). Die polymorphie des nicotinsäureamids. Ber. Dtsch. Chem. Ges..

[19] Hino T, Ford JL, Powell MW (2001). Assessment of nicotinamide polymorphs by differential scanning calorimetry. Thermochim. Acta.

[20] Wright WB, King GSD (1954). The crystal structure of nicotinamide. Acta Crystallogr..

[21] Li JJ, Bourne SA, Caira MR (2011). New polymorphs of isonicotinamide and nicotinamide. Chem. Commun..

[22] Chen S, Guzei IA, Yu L (2005). New polymorphs of ROY and new record for coexisting polymorphs of solved structures. J. Am. Chem. Soc..

[23] Chen SA, Xi HM, Yu L (2005). Cross-nucleation between ROY polymorphs. J. Am. Chem. Soc..

[24] Arlin JB, Price LS, Price SL, Florence AJ (2011). A strategy for producing predicted polymorphs: catemeric carbamazepine form V. Chem. Commun..

[25] Bucar DK (2013). The curious case of (caffeine)·(benzoic acid): how heteronuclear seeding allowed the formation of an elusive cocrystal. Chem. Sci..

[26] Zeidan TA (2016). A An unprecedented case of dodecamorphism: the twelfth polymorph of aripiprazole formed by seeding with its active metabolite. CrystEngComm.

[27] Cruz-Cabeza AJ, Bernstein J (2014). Conformational polymorphism. Chem. Rev..

[28] Zhang PY (2018). Harnessing cloud architecture for crystal structure prediction calculations. Cryst. Growth Des..

[29] Perdew JP, Burke K, Ernzerhof M (1996). Generalized gradient approximation made simple. Phys. Rev. Lett..

[30] Klimes J, Bowler DR, Michaelides A (2011). Van der Waals density functionals applied to solids. Phys. Rev. B.

[31] Kresse G, Furthmüller J (1996). Efficiency of ab-initio total energy calculations for metals and semiconductors using a plane-wave basis set. Comput. Mater. Sci..

[32] Kresse G, Hafner J (1993). Ab initio molecular dynamics for liquid metals. Phys. Rev. B.

[33] Kresse G, Hafner J (1994). Ab initio molecular-dynamics simulation of the liquid-metal–amorphous-semiconductor transition in germanium. Phys. Rev. B.

[34] Reilly AM (2016). Report on the sixth blind test of organic crystal structure prediction methods. Acta Crystallogr. B.

[35] Hermann J, DiStasio RA, Tkatchenko A (2017). First-principles models for van der waals interactions in molecules and materials: concepts, theory, and applications. Chem. Rev..

[36] Hoja J, Reilly AM, Tkatchenko A (2017). First-principles modeling of molecular crystals: structures and stabilities, temperature and pressure. Wires Comput. Mol. Sci..

[37] Gushurst KS, Nyman J, Boerrigter SXM (2019). The PO13 crystal structure of ROY. CrystEngComm.

[38] Li XZ (2020). The twelfth solved structure of ROY: single crystals of Y04 grown from melt microdroplets. Cryst. Growth Des..

[39] Vasileiadis M, Kazantsev AV, Karamertzanis PG, Adjiman CS, Pantelides CC (2012). The polymorphs of ROY: application of a systematic crystal structure prediction technique. Acta Crystallogr. B.

[40] Neumann MA, de Streek JV, Fabbiani FPA, Hidber P, Grassmann O (2015). Combined crystal structure prediction and high-pressure crystallization in rational pharmaceutical polymorph screening. Nat. Commun..

[41] Taylor CR (2020). Minimizing polymorphic risk through cooperative computational and experimental exploration. J. Am. Chem. Soc..

[42] Hu CT (2019). Discovering new polymorphs of paracetamol via melt crystallization. Acta Crystallogr. Sect. A.

[43] Mahieu A (2013). On the polymorphism of griseofulvin: identification of two additional polymorphs. J. Pharm. Sci..

[44] Yu L (2010). Polymorphism in molecular solids: an extraordinary system of red, orange, and yellow crystals. Acc. Chem. Res..

[45] Lopez-Mejias V, Kampf JW, Matzger AJ (2012). Nonamorphism in flufenamic acid and a new record for a polymorphic compound with solved structures. J. Am. Chem. Soc..

[46] Becke AD (1988). Density-functional exchange-energy approximation with correct asymptotic behavior. Phys. Rev. A.

[47] Lee C, Yang WT, Parr RG (1988). Development of the colle-salvetti correlation-energy formula into a functional of the electron density. Phys. Rev. B.

[48] Frisch, M. J. et al. (Gaussian 09, Wallingford, CT, 2009).

[49] Grimme S, Antony J, Ehrlich S, Krieg HA (2010). Consistent and accurate ab initio parametrization of density functional dispersion correction (DFT-D) for the 94 elements H-Pu. J. Chem. Phys..

[50] Miwa Y, Mizuno T, Tsuchida K, Taga T, Iwata Y (1999). Experimental charge density and electrostatic potential in nicotinamide. Acta Crystallogr. B.

[51] Vogelsanger B, Brown RD, Godfrey PD, Pierlot AP (1991). The microwave spectrum of a vitamin: nicotinamide. J. Mol. Spectrosc..

[52] Bathori NB, Lemmerer A, Venter GA, Bourne SA, Caira MR (2011). Pharmaceutical co-crystals with isonicotinamide-vitamin B3, clofibric acid, and diclofenac-and two isonicotinamide hydrates. Cryst. Growth Des..

