# Supplementary material for: Rich polymorphism in nicotinamide revealed by melt crystallization and crystal structure prediction
Source: Commun Chem. 2020 Nov 4;3:152. doi: 10.1038/s42004-020-00401-1 (PMC9814109; doi:10.1038/s42004-020-00401-1)
Supplement: Supplementary file 1 — Supplementary Information [file 42004_2020_401_MOESM1_ESM.pdf]

Rich polymorphism in nicotinamide revealed by melt crystallization and crystal structure prediction

Xizhen Li,[‡a] Xiao Ou,[‡a] Bingquan Wang,[‡a] Haowei Rong,[a] Bing Wang,[b] Chao Chang,[b] Baimei Shi,[b] Lian Yu,[c] Ming Lu [*ad]

[a] School of Pharmaceutical Sciences, Sun Yat-sen University, Guangzhou 510006, China

[b] XtalPi Inc., Shenzhen Jingtai Technology Co., Ltd., Shenzhen 518100, China

[c] School of Pharmacy, University of Wisconsin – Madison, Madison, Wisconsin, USA

[d] Guangdong Provincial Key Laboratory of New Drug Design and Evaluation, Sun Yat-sen University, Guangzhou 510006, China

[‡] These authors contributed equally

[*] Corresponding author, Email: luming3@mail.sysu.edu.cn

Electronic Supplementary Information

Content

1. Powder X-ray Diffraction (PXRD)..3

2. Raman Microscopy..4

3. Fourier Transform Infrared (FTIR) Spectroscopy........................... 5

4. Cross-nucleation among Forms γ, δ and ζ................................... 6

5. Formation of Form ι *via* Pseudoseeding......................................7

6. Single-crystal Structure Determination.. 7

7. Crystal Structure Prediction (CSP)..11

8. Thermal Stability and Polymorphic Conversion.........................19

9. Supplementary Method..25

1. Powder X-ray Diffraction (PXRD)

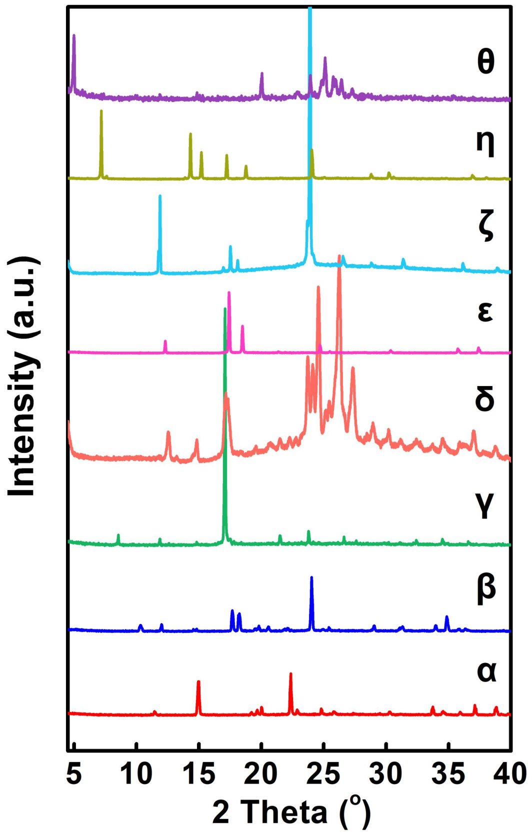

Supplementary Figure 1. PXRD patterns of the NIC polymorphs

2. Raman Microscopy

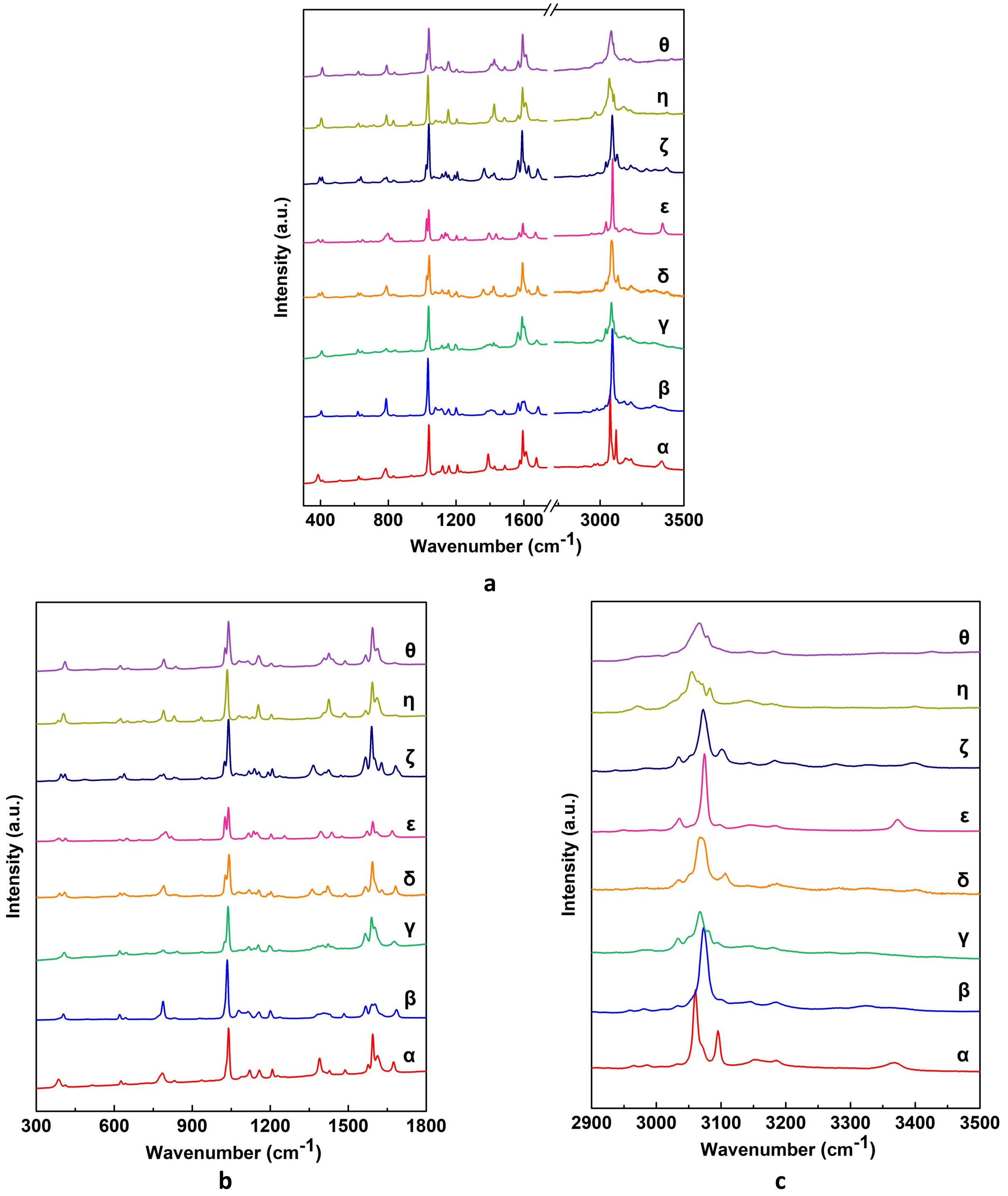

Supplementary Figure 2. Raman spectra of NIC polymorphs. (a) 300-3500 cm^{-1}; (b) 300-1800 cm^{-1}; (c) 2900-3500 cm^{-1}

3. Fourier Transform Infrared (FTIR) Spectroscopy

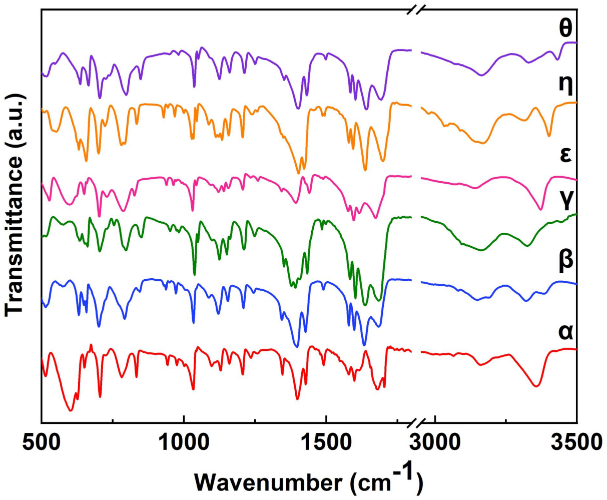

a

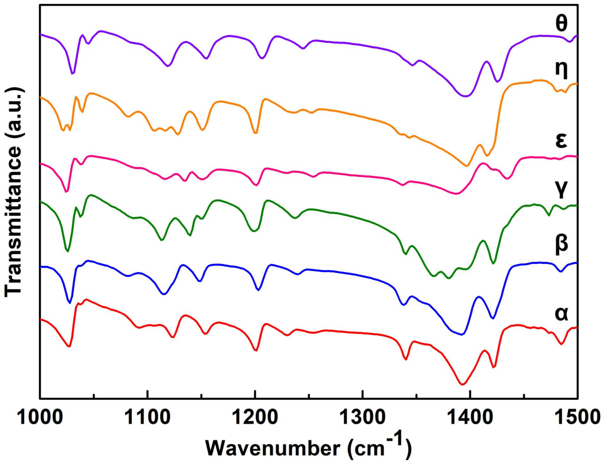

b

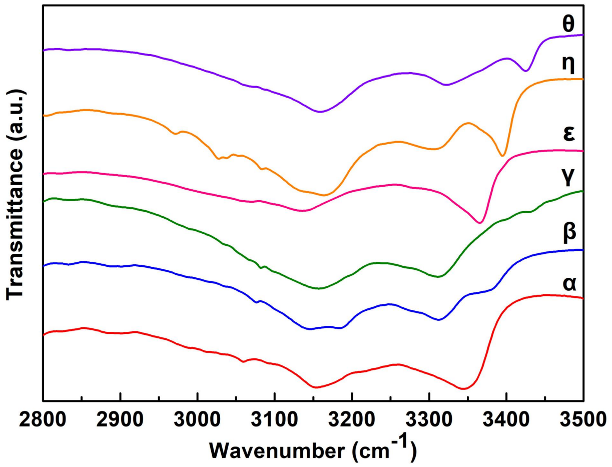

c

Supplementary Figure 3. FTIR spectra of NIC polymorphs. (a) 500-3500 cm^{-1}; (b) 1000-1500 cm^{-1}; (c) 2800-3500 cm^{-1}

4. Cross-nucleation among Forms γ, δ and ζ

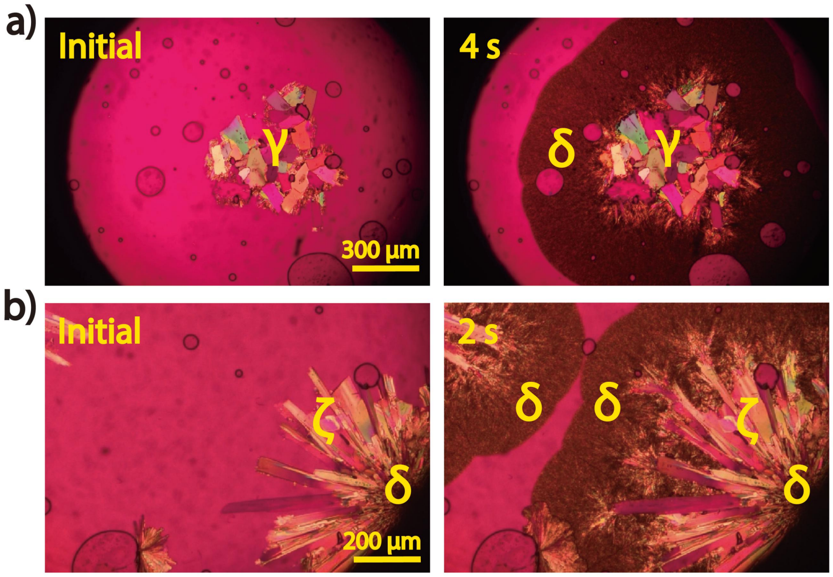

Supplementary Figure 4. **Cross-nucleation of Form δ on the growth fronts of Forms γ and ζ at 70 °C.** (a) Seeds of Form γ were prepared first and then quenched at 70 °C to cross-nucleate Form δ. (b) A NIC melt was quenched at room temperature to nucleate Form δ and then was transferred to 105 °C for cross-nucleating Form ζ. This sample was quenched at 70 °C to trigger the cross-nucleation of Form δ on the surface of Form ζ

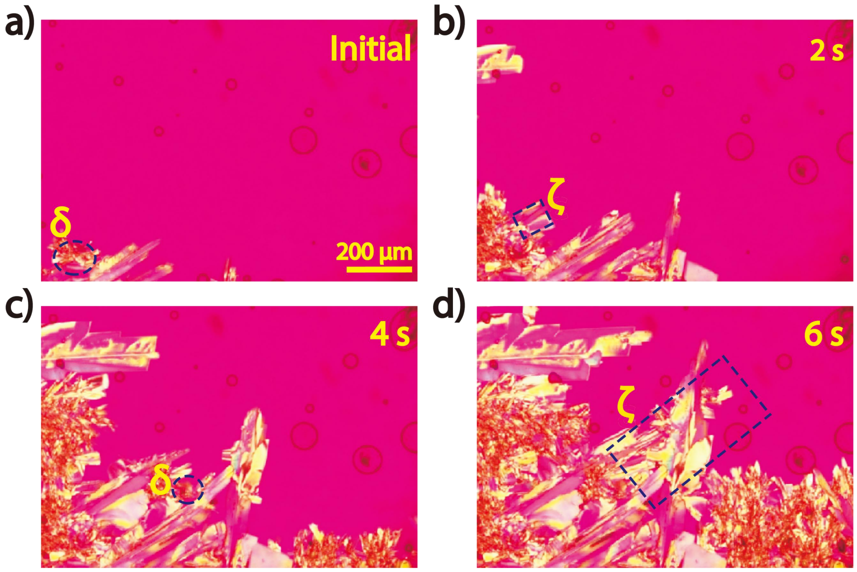

Supplementary Figure 5. Forms δ and ζ interactively cross-nucleate on each other at 95 °C. (a) Seeds of Form δ was transferred to 95 °C; (b) Form ζ cross-nucleated on the growth front of Form δ after 2 s; (c) Form δ nucleated on the surface of newly-grown Form ζ after 4 s; (d) Form ζ nucleated and grew on the surface of Form δ after 6 s

5. Formation of Form ι *via* Pseudoseeding

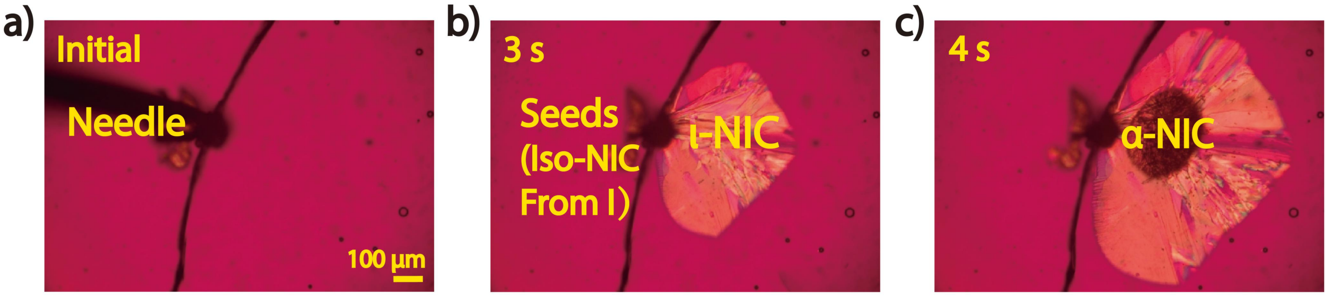

Supplementary Figure 6. Formation of Form ι and ι-to-α phase conversion at 90 °C. (a) Iso-nicotinamide (iso-NIC) Form I was seeded on the edge of the nicotinamide (NIC) melt. (b) NIC Form ι nulceated on iso-NIC seeds; (c) NIC Form ι rapidly converted to Form α

6. Single-crystal Structure Determination

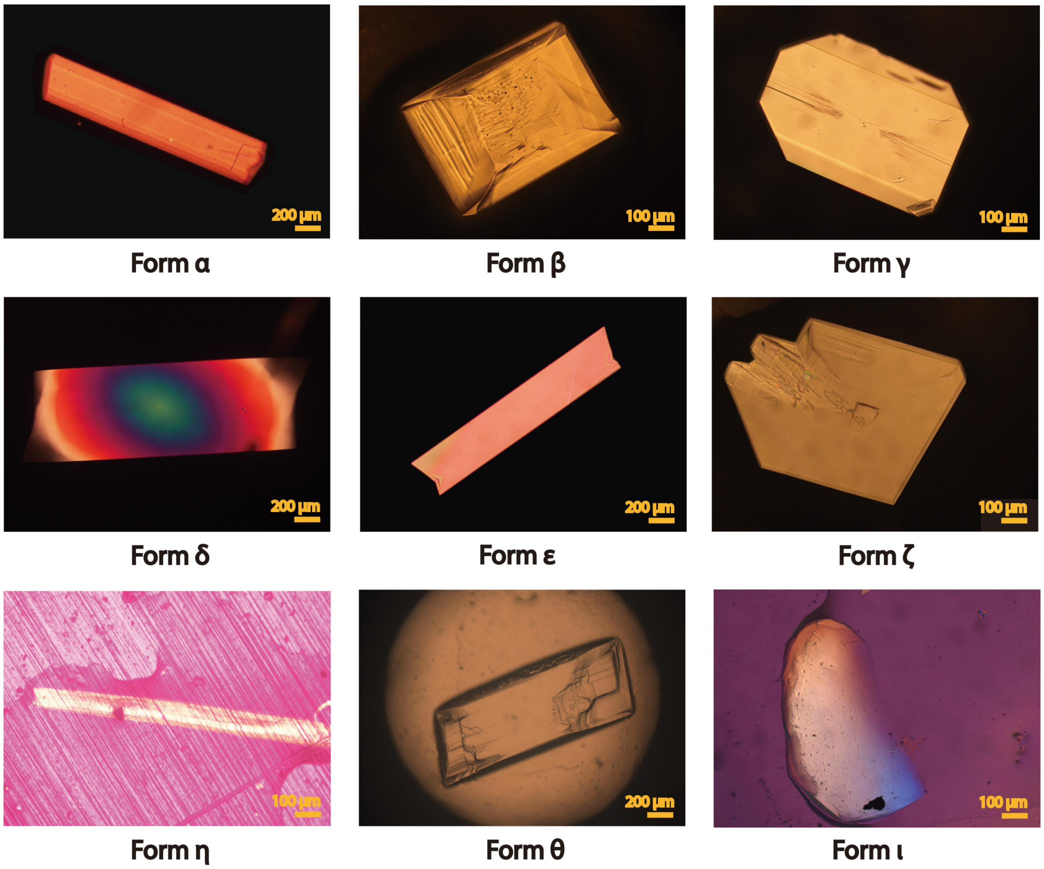

Supplementary Figure 7. **Single crystals of NIC polymorphs grown from melt microdroplets**

Supplementary Table 1. Cultivation conditions of NIC single crystals

Polymorph	Partial melting temperature (°C)	Cultivation temperature (°C)	$T_{Cultivation}/T_{Melting}$
α	129	127	0.995
β	116.5	110	0.982
γ	115	110	0.987
δ	114	108.5	0.986
ε	110.5	108	0.993
ζ	109.5	108	0.996
η	108	106	0.995
θ	104.5	102	0.992
ι	103	95	0.979

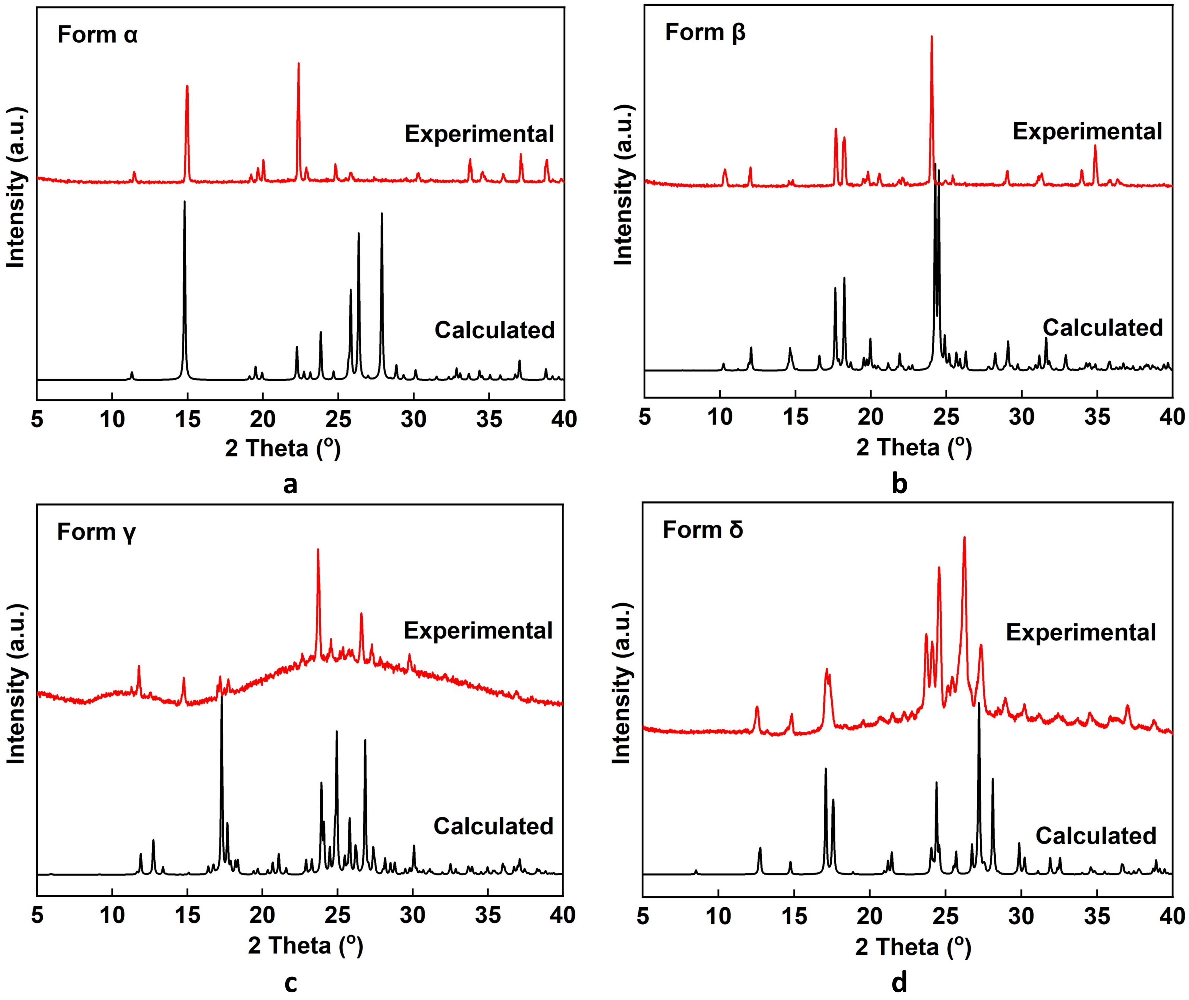

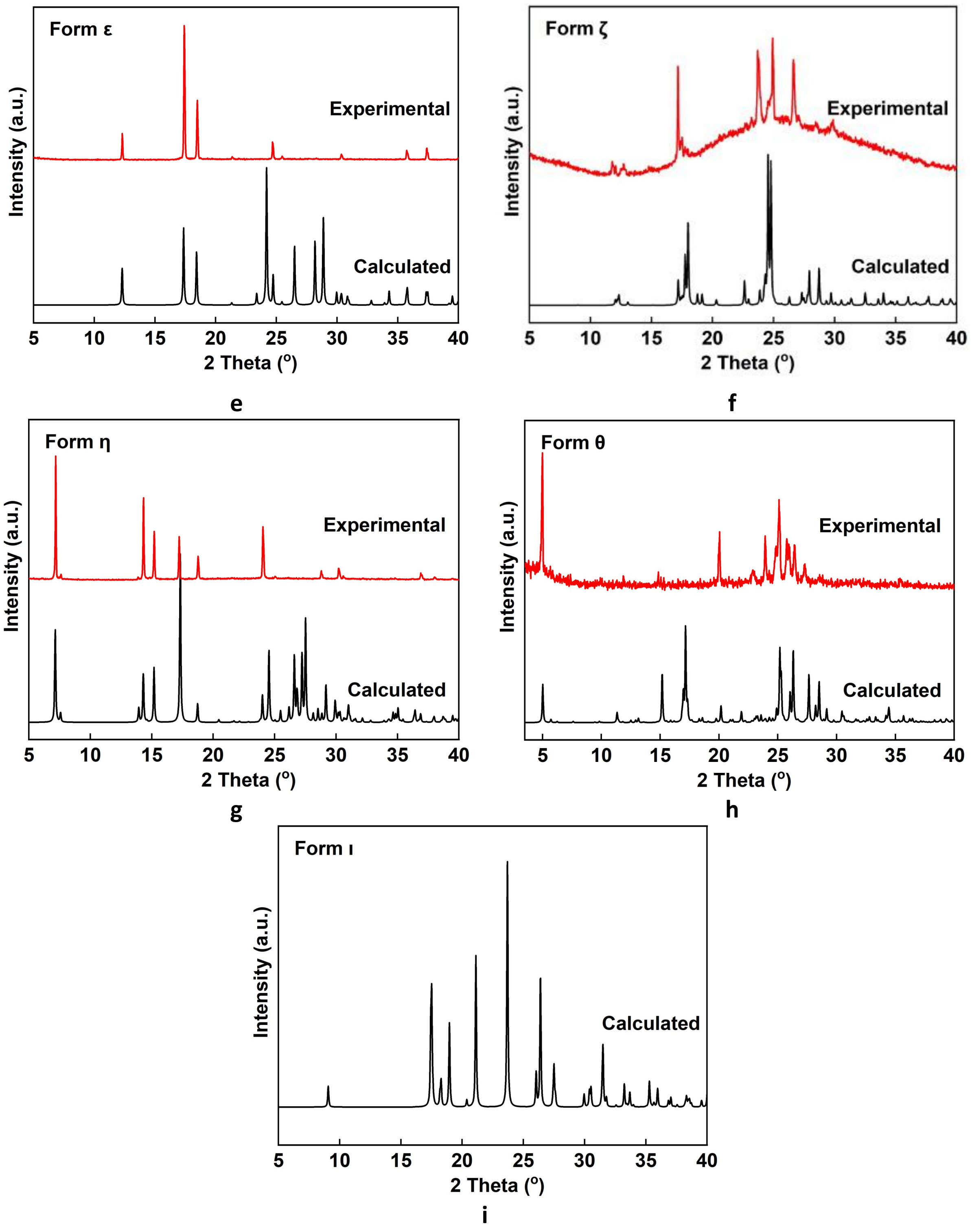

Supplementary Figure 8. Calculated and experimental PXRD patterns of NIC polymorphs. (a) Form α; (b) Form β; (c) Form γ; (d) Form δ; (e) Form ε; (f) Form ζ; (g) Form η; (h) Form θ; (i) Form ι

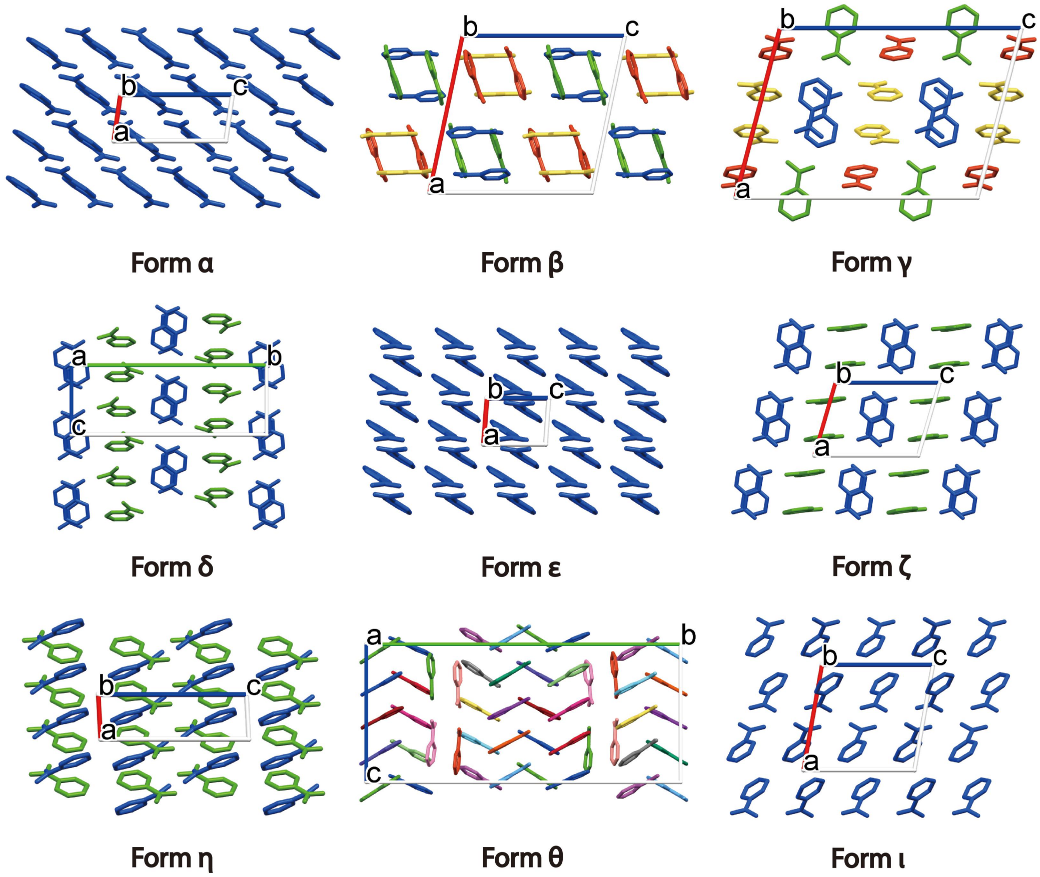

Supplementary Figure 9. Crystal packing diagrams of NIC polymorphs. All structures are shown along the *b*-axis, except for Forms δ and θ, where the structures are shown along the *a*-axis. Different colors are used to indicate conformationally distinct molecules

Supplementary Table 2. **Torsion angle ϑ (C5-C4-C6-N2') in 37 NIC conformations**

Polymorph	ϑ (C5-C4-C6-N2)			
	Mol. 1	Mol. 2	Mol. 3	Mol. 4
α	-23.1	-	-	-
β	12.6	-8.7	-23.1	-15.6
γ	0.6	-175.1	15.5	178.2
δ	0.1	160.9	-	-
ε	-151.5	-	-	-
ζ	1.0	-175.3	-	-
η	25.4	-3.2	-	-
θ	-6.0	0.9	7.3	-176.7
	-171.0	2.8	-5.3	173.1
	-5.1	-169.1	-14.4	176.3
	4.0	4.5	0.9	8.2
	-3.6	-4.3	167.7	-2.6
ι	-28.8	-	-	-

7. Crystal Structure Prediction (CSP)

Supplementary Table 3. Details of predicted low-energy structures of NIC

Rank	Relative Lattice Energy (kJ/mol)	Cell Dimensions						Density (g/cm³)	Z'	Space Group	Conformer Type*	Corresponding Polymorph
		a (Å)	b (Å)	c (Å)	α	ρ	γ					
**1**	0	3.925	15.217	9.475	90.000	97.211	90.000	1.445	1	P21/c (14)	I	α
**2**	0.53	4.489	28.482	8.941	90.000	87.367	90.000	1.421	2	P21/c (14)	I, I	-
**3**	1.1	12.969	5.109	17.111	90.000	92.998	90.000	1.433	2	P21/c (14)	I, I	-
**4**	1.34	3.881	10.299	19.458	90.000	47.769	90.000	1.409	1	P21/c (14)	II	-
**5**	1.45	9.962	5.100	14.042	90.000	127.225	90.000	1.428	1	P21/c (14)	I	-
**6**	1.47	13.183	12.193	3.762	93.036	94.538	71.330	1.421	2	P-1 (2)	I, I	η
**7**	1.49	7.399	20.949	7.342	90.000	90.249	90.000	1.426	2	P21/c (14)	I, II	δ
**8**	1.73	5.174	14.346	3.814	90.000	86.075	90.000	1.436	1	P21 (4)	II	ε
**9**	2.22	5.058	5.735	20.134	90.000	77.909	90.000	1.42	1	P21/c (14)	II	-
**10**	2.37	8.117	5.044	13.955	90.000	96.975	90.000	1.43	1	P21/c (14)	I	-
**11**	2.39	9.996	6.066	9.884	90.000	101.461	90.000	1.381	1	P21/c (14)	I	ι
**12**	2.57	3.686	12.941	12.654	104.621	89.252	95.395	1.395	2	P-1 (2)	I, I	-
**13**	2.70	5.652	20.033	5.067	84.828	88.279	89.495	1.42	2	P-1 (2)	I, II	-
**14**	2.72	7.518	7.235	24.531	90.000	120.218	90.000	1.407	2	P21/c (14)	II, II	-

**15**	2.83	14.980	3.884	10.311	90.000	108.918	90.000	1.429	1	P21/c (14)	I	-
**16**	2.97	13.737	3.916	10.566	90.000	92.663	90.000	1.429	1	P21/c (14)	I	-
**17**	3.08	5.101	5.669	20.042	90.000	94.097	90.000	1.403	1	P21/c (14)	I	-
**18**	3.11	3.776	28.797	6.696	90.000	129.034	90.000	1.434	1	P21/c (14)	II	-
**19**	3.14	8.040	5.060	30.382	90.000	112.595	90.000	1.422	2	P21/c (14)	I, I	-
**20**	3.14	15.066	5.018	17.468	90.000	58.847	90.000	1.436	2	P21/c (14)	I, I	-
**21**	3.27	28.189	3.890	10.422	90.000	89.044	90.000	1.42	2	P21/c (14)	I, II	-
**22**	3.31	18.082	7.139	8.968	90.000	91.632	90.000	1.402	2	P21/c (14)	I, I	-
**23**	3.37	9.882	5.099	22.885	90.000	97.068	90.000	1.418	2	P21/c (14)	I, I	-
**24**	3.42	5.034	5.488	20.343	90.000	90.000	90.000	1.443	1	P212121 (19)	II	-
**25**	3.44	6.284	14.218	8.333	90.000	128.530	90.000	1.393	1	P21/c (14)	I	-
**26**	3.45	7.700	7.436	11.011	90.000	67.642	90.000	1.391	1	P21/c (14)	I	-
**27**	3.48	14.518	5.166	3.879	97.019	84.483	91.142	1.411	1	P-1 (2)	I	-
**28**	3.48	11.247	5.125	9.983	86.839	97.710	89.975	1.425	2	P-1 (2)	I, II	-
**29**	3.49	13.954	5.136	16.037	90.000	83.318	90.000	1.421	2	P21/c (14)	I, II	-
**30**	3.5	5.086	5.676	19.904	90.000	90.000	90.000	1.412	1	P212121 (19)	I	-
**31**	3.51	20.294	5.080	5.487	90.000	89.914	90.000	1.434	2	P21 (4)	I, II	-
**32**	3.54	5.390	5.070	20.737	90.000	91.638	90.000	1.432	1	P21/c (14)	II	-
**33**	3.55	5.098	14.493	18.125	90.000	121.317	90.000	1.418	2	P21/c (14)	II, II	-
**34**	3.57	8.704	5.100	12.869	87.806	86.431	87.132	1.425	2	P-1 (2)	I, I	-

**35**	3.59	11.544	5.047	19.792	90.000	82.202	90.000	1.42	2	P21/c (14)	I, I	-
**36**	3.76	3.809	5.155	29.070	89.643	90.164	95.525	1.428	2	P-1 (2)	I, II	-
**37**	3.8	14.983	3.833	9.914	90.000	90.000	90.000	1.425	1	Pca21 (29)	II	-
**38**	3.83	31.732	5.214	16.636	90.000	123.874	90.000	1.42	2	C2/c (15)	I, II	-
**39**	3.84	15.235	3.956	20.923	90.000	64.748	90.000	1.422	2	P21/c (14)	I, I	-
**40**	3.89	9.909	5.130	22.565	90.000	97.083	90.000	1.425	2	P21/c (14)	I, II	-
**41**	3.89	17.008	5.144	13.824	90.000	107.953	90.000	1.41	2	P21/c (14)	I, II	-
**42**	4	14.078	3.910	20.803	90.000	85.113	90.000	1.422	2	P21/c (14)	I, I	-
**43**	4.02	15.219	5.063	16.179	90.000	66.267	90.000	1.422	2	P21/c (14)	I, I	-
**44**	4.06	17.704	6.741	9.834	90.000	91.279	90.000	1.383	2	P21/c (14)	I, II	-
**45**	4.08	8.899	6.803	9.745	90.000	91.420	90.000	1.375	1	P21/c (14)	II	-
**46**	4.1	5.124	11.270	11.206	66.704	83.063	91.151	1.379	2	P-1 (2)	I, I	-
**47**	4.1	28.406	3.860	10.404	90.000	93.134	90.000	1.424	2	P21/c (14)	I, II	-
**48**	4.12	8.783	12.957	5.167	98.157	101.024	87.672	1.42	2	P-1 (2)	I, II	-
**49**	4.13	28.751	3.804	5.216	84.551	92.895	91.187	1.43	2	P-1 (2)	II, II	-
**50**	4.14	5.196	28.733	3.826	93.328	85.156	88.425	1.428	2	P-1 (2)	I, II	-
**51**	4.2	29.483	3.803	10.226	90.000	99.407	90.000	1.434	2	P21/c (14)	II, II	-
**52**	4.21	20.760	5.431	10.432	90.000	99.739	90.000	1.399	2	P21/c (14)	II, II	-
**53**	4.21	5.170	29.159	3.805	90.000	96.167	90.000	1.423	2	P21 (4)	I, II	-
**54**	4.22	29.201	3.781	5.179	94.902	87.861	91.252	1.425	2	P-1 (2)	II, II	-

**55**	4.22	7.035	5.087	16.183	90.000	97.923	90.000	1.414	1	P21/c (14)	I	-
**56**	4.24	8.466	13.091	5.178	88.478	86.437	86.553	1.419	2	P-1 (2)	II, II	-
**57**	4.26	14.583	3.869	10.443	90.000	75.728	90.000	1.421	1	P21/c (14)	II	-
**58**	4.27	27.938	3.922	10.530	90.000	96.260	90.000	1.415	2	P21/c (14)	I, II	-
**59**	4.28	35.570	3.762	20.968	90.000	125.528	90.000	1.421	2	C2/c (15)	II, II	-
**60**	4.31	8.367	5.182	13.437	92.682	95.340	96.109	1.409	2	P-1 (2)	I, II	-
**61**	4.32	12.951	5.088	8.669	90.000	86.264	90.000	1.423	2	P21 (4)	I, I	-
**62**	4.33	7.948	8.151	10.099	80.733	106.920	75.803	1.381	2	P-1 (2)	I, II	-
**63**	4.34	8.418	6.900	9.953	90.000	92.028	90.000	1.404	1	P21/c (14)	I	-
**64**	4.35	15.374	5.121	15.891	90.000	113.228	90.000	1.411	2	P21/c (14)	I, I	-
**65**	4.39	29.043	3.824	10.336	90.000	81.062	90.000	1.431	2	P21/c (14)	I, II	-
**66**	4.41	14.750	3.898	20.770	90.000	73.221	90.000	1.419	2	P21/c (14)	I, I	-
**67**	4.42	20.816	5.815	10.003	90.000	103.182	90.000	1.376	2	P21/c (14)	I, II	-
**68**	4.44	8.975	7.036	9.209	90.000	92.232	90.000	1.396	1	P21/c (14)	I	-
**69**	4.45	6.911	16.205	5.115	89.478	87.535	96.469	1.427	2	P-1 (2)	I, II	-
**70**	4.45	5.010	14.345	8.125	90.000	91.026	90.000	1.389	1	P21/c (14)	I	-
**71**	4.46	5.043	14.544	7.900	90.000	91.377	90.000	1.4	1	P21/c (14)	II	-
**72**	4.48	5.239	28.649	3.811	88.174	84.994	84.958	1.43	2	P-1 (2)	I, II	-
**73**	4.48	3.838	5.286	14.364	82.405	89.350	82.293	1.417	1	P-1 (2)	I	-
**74**	4.48	29.065	3.830	10.299	90.000	82.461	90.000	1.427	2	P21/c (14)	I, II	-

**75**	4.49	28.521	5.231	3.905	98.848	88.113	85.176	1.416	2	P-1 (2)	I, II	-
**76**	4.5	29.028	3.820	10.425	90.000	79.863	90.000	1.425	2	P21/c (14)	II, II	-
**77**	4.51	8.557	5.100	26.307	90.000	84.959	90.000	1.419	2	P21/c (14)	I, I	-
**78**	4.51	15.180	5.161	16.975	90.000	120.133	90.000	1.41	2	P21/c (14)	II, II	-
**79**	4.51	13.973	5.099	20.079	90.000	53.116	90.000	1.418	2	P21/c (14)	I, II	-
**80**	4.52	8.552	5.203	31.202	90.000	55.229	90.000	1.423	2	P21/c (14)	II, II	-
**81**	4.53	27.679	3.949	10.534	90.000	95.199	90.000	1.415	2	P21/c (14)	I, II	-
**82**	4.56	6.828	5.162	16.206	90.000	95.107	90.000	1.426	1	P21/c (14)	II	-
**83**	4.56	8.202	5.122	27.533	90.000	96.027	90.000	1.41	2	P21/c (14)	I, II	-
**84**	4.56	13.884	5.096	8.141	90.000	96.597	90.000	1.417	2	P21 (4)	I, II	-
**85**	4.57	30.774	3.819	10.100	90.000	107.202	90.000	1.431	1	C2/c (15)	II	-
**86**	4.6	28.326	5.294	3.857	98.840	88.032	88.666	1.421	2	P-1 (2)	I, II	-
**87**	4.61	11.421	5.076	20.099	90.000	83.614	90.000	1.401	2	P21/c (14)	I, I	-
**88**	4.62	8.527	5.217	13.373	81.145	90.638	78.471	1.41	2	P-1 (2)	II, II	-
**89**	4.62	20.204	5.133	22.102	90.000	98.464	90.000	1.431	2	C2/c (15)	I, II	-
**90**	4.64	15.278	5.141	15.990	90.000	66.419	90.000	1.41	2	P21/c (14)	I, II	-
**91**	4.66	10.471	5.157	11.522	90.000	66.525	90.000	1.421	2	P21 (4)	I, II	-
**92**	4.69	10.547	8.056	8.030	107.755	99.302	108.692	1.376	2	P-1 (2)	I, II	ζ
**93**	4.72	29.119	3.797	10.387	90.000	80.979	90.000	1.43	2	P21/c (14)	I, II	-
**94**	4.74	3.763	5.212	14.554	94.434	92.416	96.178	1.435	1	P-1 (2)	II	-

**95**	4.77	34.668	5.130	13.482	90.000	108.386	90.000	1.426	2	C2/c (15)	I, II	-
**96**	4.77	9.169	5.117	30.234	90.000	126.232	90.000	1.418	2	P21/c (14)	II, II	-
**97**	4.77	22.964	10.050	5.083	90.000	90.000	90.000	1.383	2	P212121 (19)	I, I	-
**98**	4.77	11.322	5.082	20.043	90.000	81.203	90.000	1.424	2	P21/c (14)	I, II	-
**99**	4.77	29.023	5.176	3.817	96.414	91.562	91.304	1.425	2	P-1 (2)	I, II	-
**100**	4.79	13.872	5.200	20.304	90.000	52.013	90.000	1.406	2	P21/c (14)	I, II	-
**101**	4.79	14.490	4.083	10.439	90.000	109.979	90.000	1.398	1	P21/c (14)	I	-
**102**	4.79	15.612	3.788	20.978	90.000	114.430	90.000	1.436	2	P21/c (14)	II, II	-
**103**	4.8	8.604	5.091	30.197	90.000	120.114	90.000	1.418	2	P21/c (14)	I, I	-
**104**	4.84	9.951	6.800	20.121	90.000	59.575	90.000	1.382	2	P21/c (14)	I, I	-
**105**	4.84	27.331	4.202	10.317	90.000	79.431	90.000	1.393	2	P21/c (14)	I, I	-
**106**	4.85	13.772	6.454	6.796	98.306	91.531	83.758	1.365	2	P-1 (2)	II, II	-
**107**	4.85	12.920	5.099	17.353	90.000	93.711	90.000	1.422	2	P21/c (14)	I, I	-
**108**	4.86	3.887	29.301	5.129	90.000	96.967	90.000	1.399	1	P21/c (14)	I	-
**109**	4.86	7.487	5.149	38.496	90.000	129.130	90.000	1.409	2	P21/c (14)	II, II	-
**110**	4.89	14.698	3.846	10.064	90.000	93.193	90.000	1.428	1	P21/c (14)	II	-
**111**	4.89	8.556	13.362	5.234	89.892	76.551	93.129	1.396	2	P-1 (2)	I, II	-
**112**	4.9	27.869	3.885	10.581	90.000	94.882	90.000	1.421	2	P21/c (14)	I, I	-
**113**	4.91	16.446	3.894	21.090	90.000	122.398	90.000	1.423	2	P21/c (14)	I, I	-
**114**	4.91	5.277	14.414	3.896	86.837	80.386	99.677	1.413	1	P-1 (2)	II	-

**115**	4.91	7.311	10.764	7.983	69.878	83.423	96.205	1.402	2	P-1 (2)	I, II	-
**116**	4.92	28.114	3.860	10.573	90.000	96.133	90.000	1.422	2	P21/c (14)	I, II	-
**117**	4.93	10.381	5.145	21.314	90.000	93.127	90.000	1.427	2	P21/c (14)	II, II	-
**118**	4.94	8.453	13.419	5.207	79.269	96.926	94.814	1.411	2	P-1 (2)	I, II	-
**119**	4.94	13.644	5.121	20.221	90.000	53.524	90.000	1.428	2	P21/c (14)	I, II	-
**120**	4.94	3.797	29.395	5.173	94.289	96.574	91.178	1.419	2	P-1 (2)	I, II	-
**121**	4.96	5.086	6.906	16.661	90.000	97.346	90.000	1.398	1	P21/c (14)	I	-
**122**	4.97	13.686	5.123	16.347	90.000	95.854	90.000	1.423	2	P21/c (14)	I, II	-
**123**	4.97	26.726	5.145	16.459	90.000	94.095	90.000	1.437	2	C2/c (15)	I, II	-
**124**	4.98	18.546	6.838	9.314	90.000	90.790	90.000	1.374	2	P21/c (14)	I, II	-

* The conformers were divided into two types according to the value of the torsion angle θ (C5-C4-C6-N2’) : type I refers to the conformers with -45° < θ < 45°, and type II refers to the conformers with 135° < θ ≤ 180° or -180° ≤ θ < -135°.

Supplementary Table 4. Comparison of the predicted structures with the experimentally observed structures of NIC

Structure	RMSD15 (Å)	Relative Lattice Energy (kJ/mol)	Rank	Z'
Form α	0.228	0	1	1
Form β	0.077	3.09	NI*	4
Form γ	0.045	2.03	NI*	4
Form δ	0.105	1.49	7	2
Form ε	0.107	1.73	8	1
Form ζ	0.811	4.69	92	2
Form η	0.135	1.47	6	2
Form θ	0.038	2.61	NI*	20
Form ι	0.202	2.39	11	1

*NI: The structure was not included in this CSP search.

8. Thermal Stability and Polymorphic Conversion

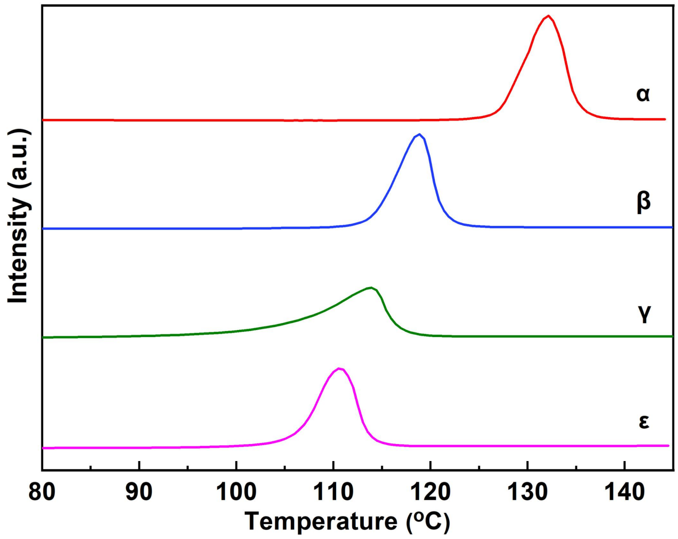

Supplementary Figure 10. **Differential scanning calorimetry (DSC) curves of four NIC polymorphs.** All measurements were performed at a heating rate of 10 °C/min (*n*=4)

Supplementary Table 5. **Melting point (T_m) and melting enthalpy (ΔH_m) of NIC polymorphs.** The values of T_m and ΔH_m were determined by using DSC at a heating rate of 10 °C/min (*n*=4). T_m values were also measured by using hot-stage combined with a polarized optical microscopy (POM)

Crystal form	$T_{m,peak(DSC)}$ (°C)	ΔH_m (kJ/mol)	$T_{m\ (POM)}$(°C)
α	131.9±0.2	20.0±0.7	129
β	118.9±0.3	17.5±0.4	116.5
γ	113.8±0.1	17.0±0.1	115
δ	-	-	114
ε	110.6±0.1	16.4±0.1	110.5
ζ	-	-	109.5
η	-	-	108
θ	-	-	104.5
ι	-	-	103

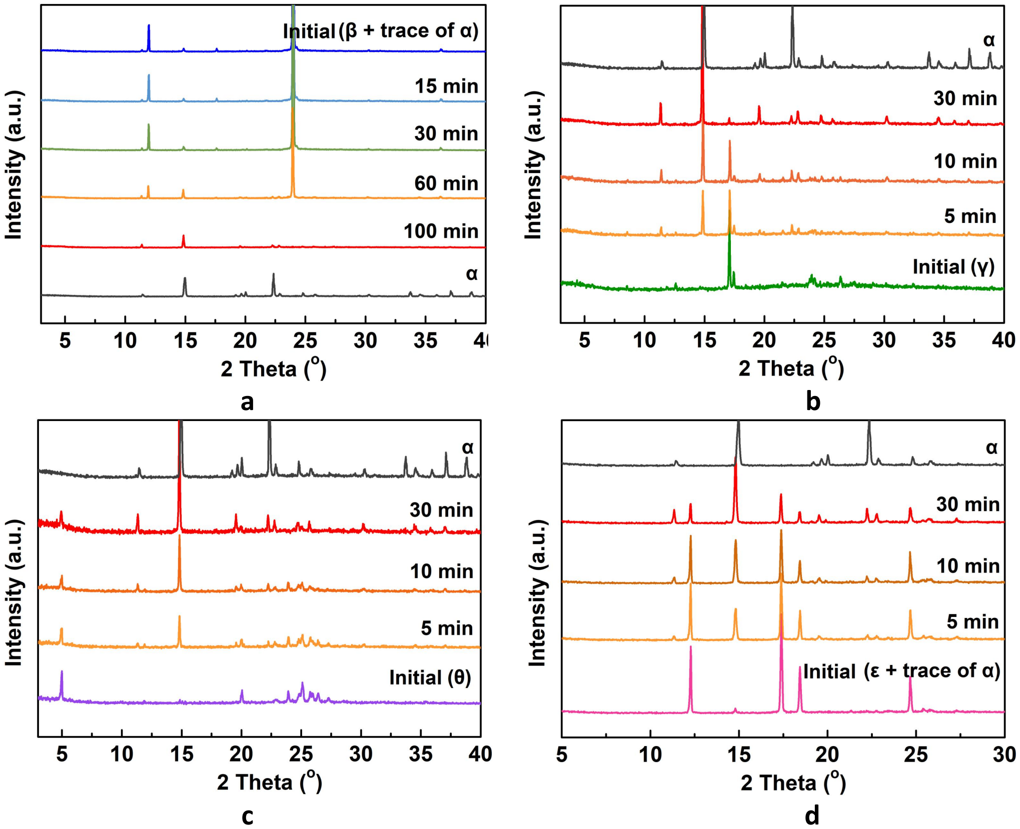

Supplementary Figure 11. PXRD patterns of the polymorphic conversion from metastable polymorphs to stable polymorph α. (a) β-to-α phase conversion; (b) γ-to-α phase conversion; (c) θ-to-α phase conversion; (d) ε-to-α phase conversion

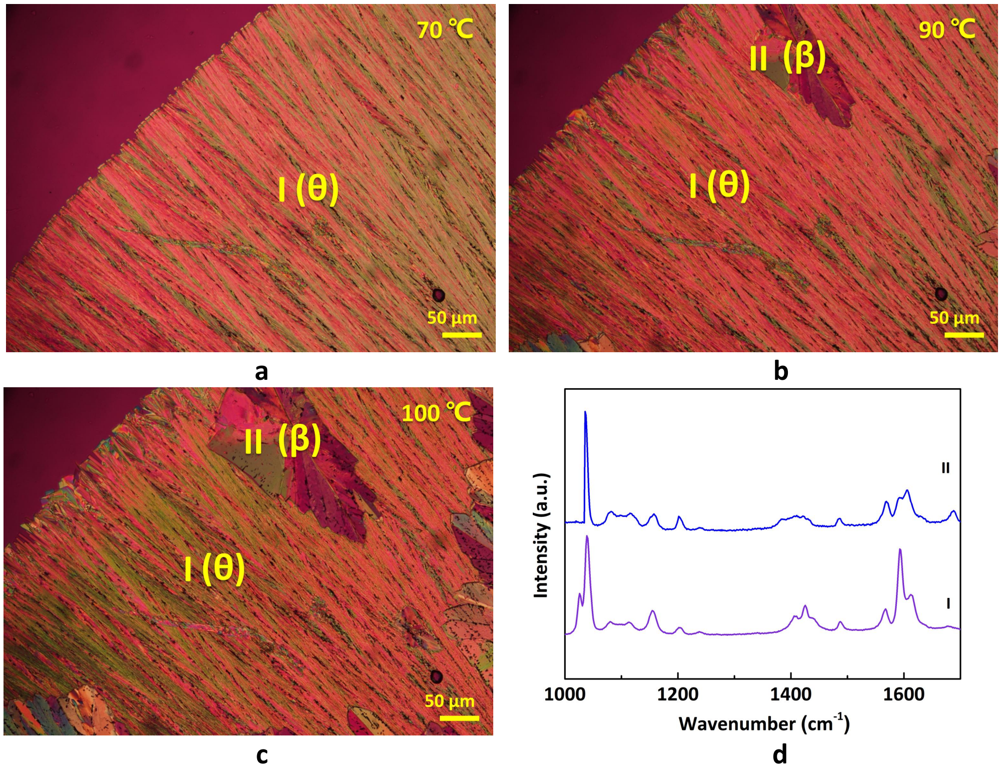

Supplementary Figure 12. Polymorphic conversion from Form θ to Form β. (a-c) POM images; (d) Raman spectra.

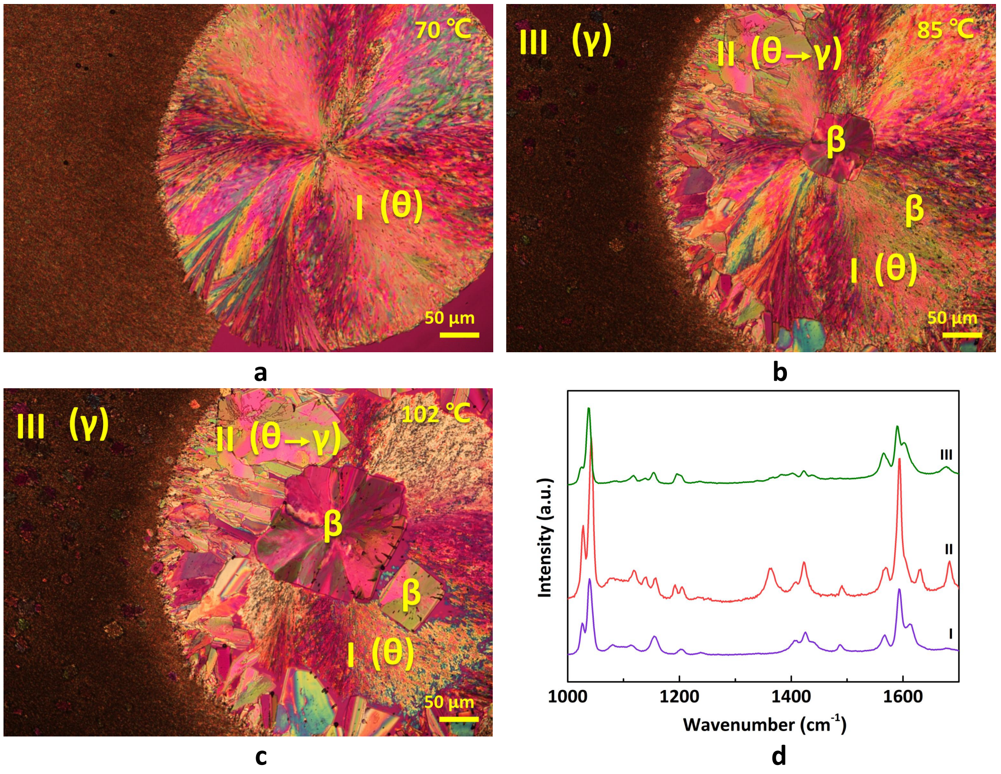

Supplementary Figure 13. Polymorphic conversion from Form θ to Form γ. (a-c) POM images; (d) Raman spectra.

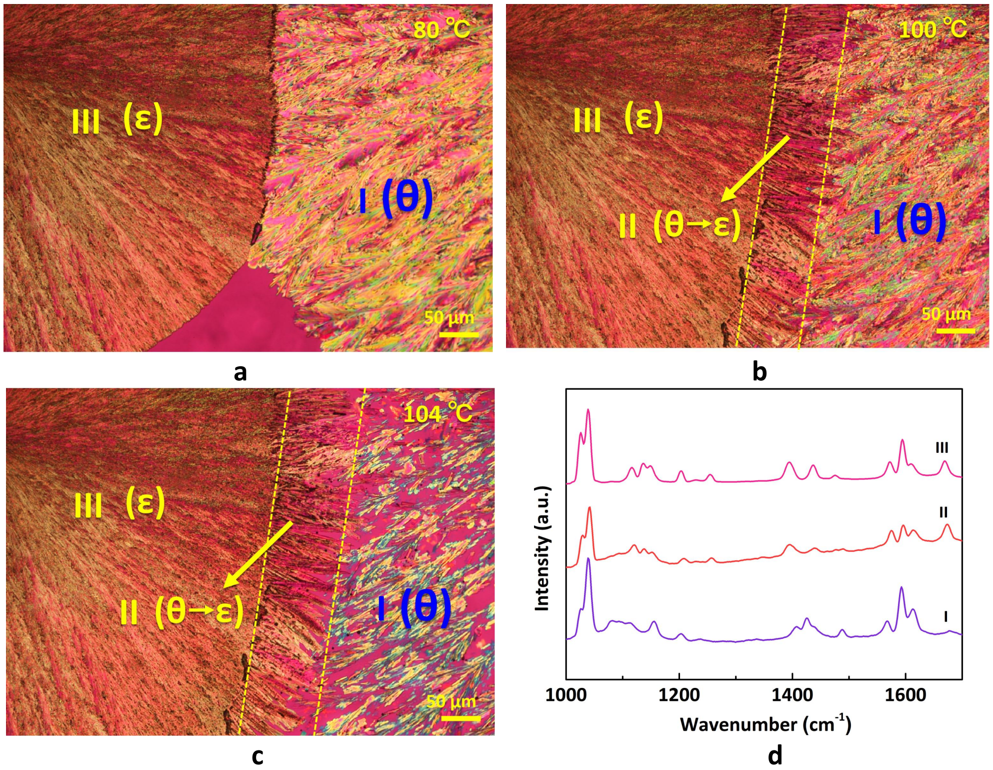

Supplementary Figure 14. Polymorphic conversion from Form θ to Form ε. (a-c) POM images; (d) Raman spectra.

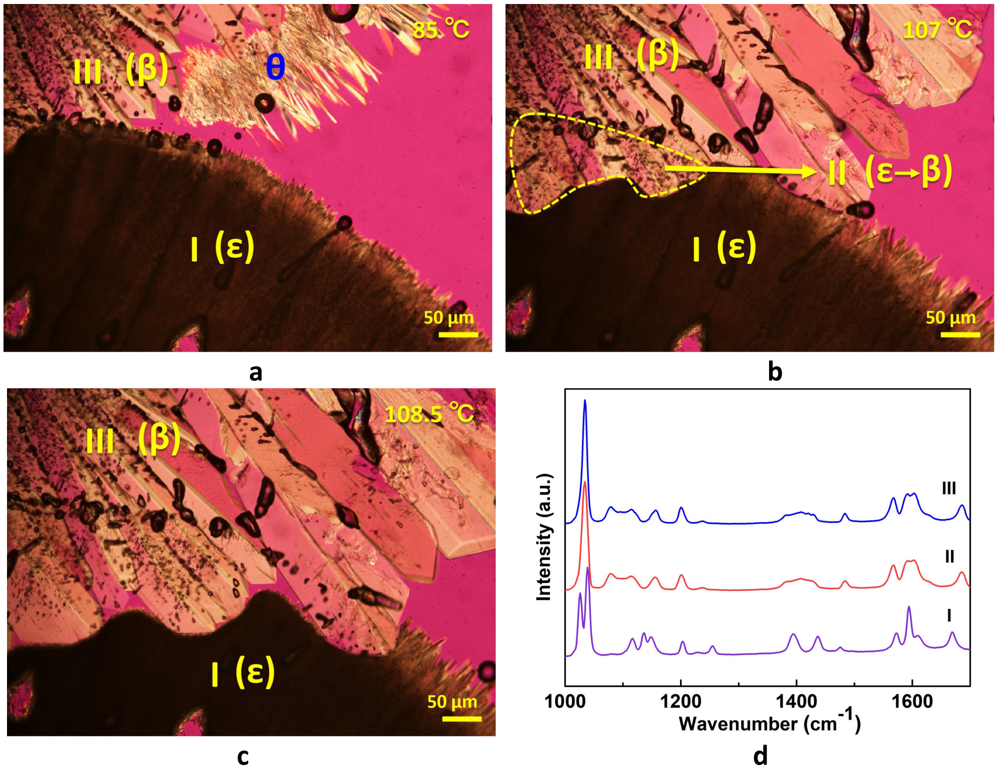

Supplementary Figure 15. Polymorphic conversion from Form ε to Form β. (a-c) POM images; (d) Raman spectra.

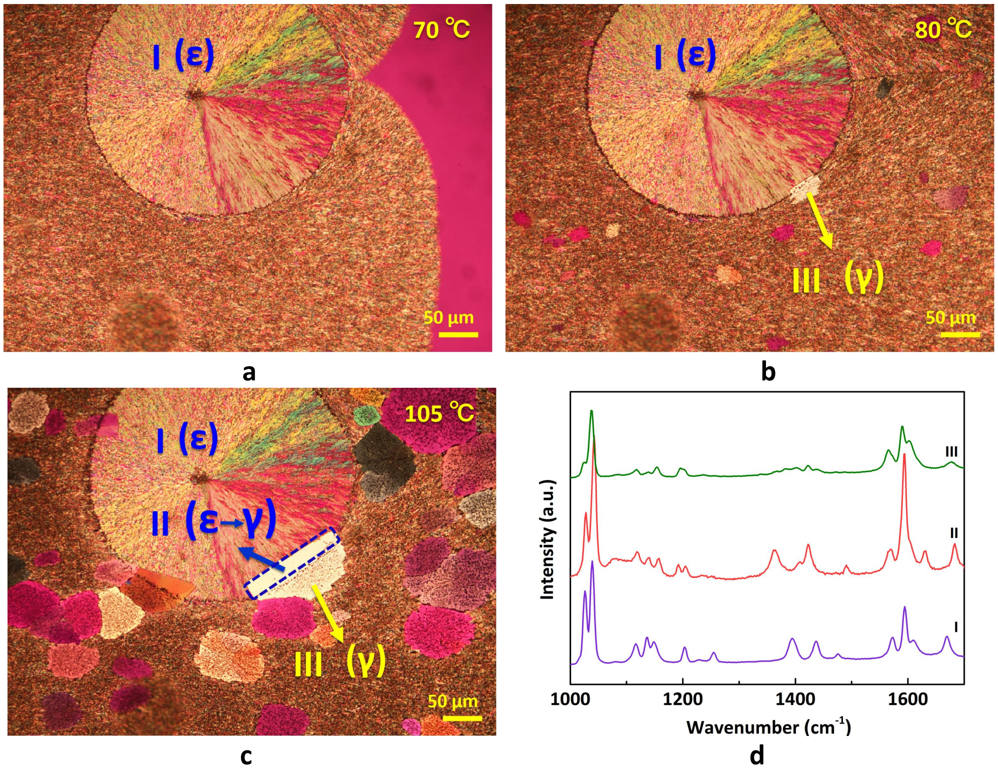

Supplementary Figure 16. Polymorphic conversion from Form ε to Form γ. (a-c) POM images; (d) Raman spectra

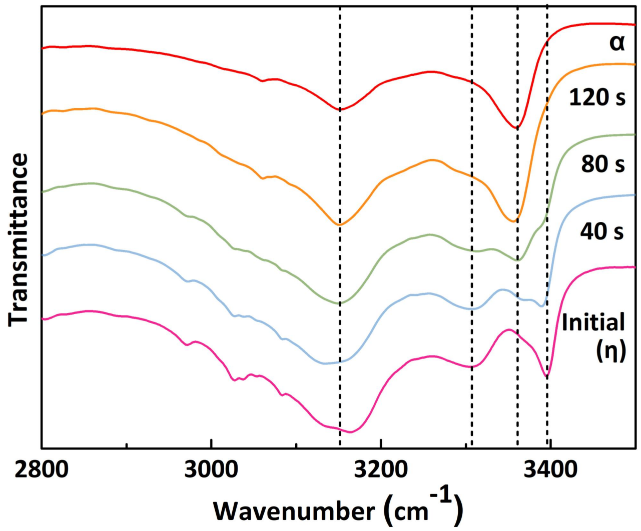

Supplementary Figure 17. Time-resolved FTIR spectra showing the η-to-α polymorphic conversion

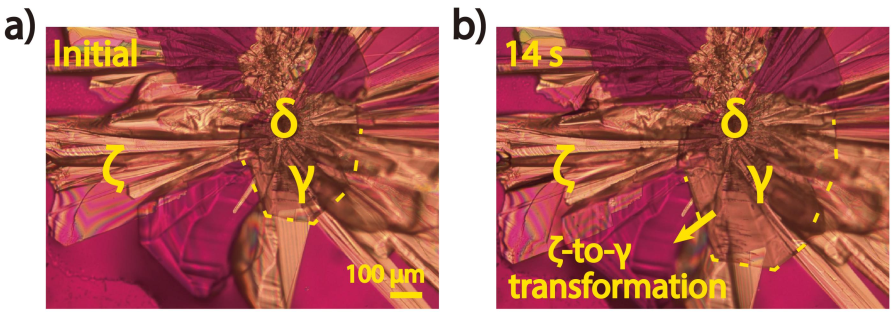

Supplementary Figure 18. Cross-nucleation of Form ζ on Form δ and the following ζ-to-γ polymorphic conversion at 105 °C. (a) A NIC melt was quenched at room temperature to nucleate Form δ and then this sample was transferred to a hot-stage preset as 105 °C to cross-nucleate Form ζ; (b) Form δ transformed to Form γ and this newly-formed Form γ triggered the ζ-to-γ polymorphic conversion

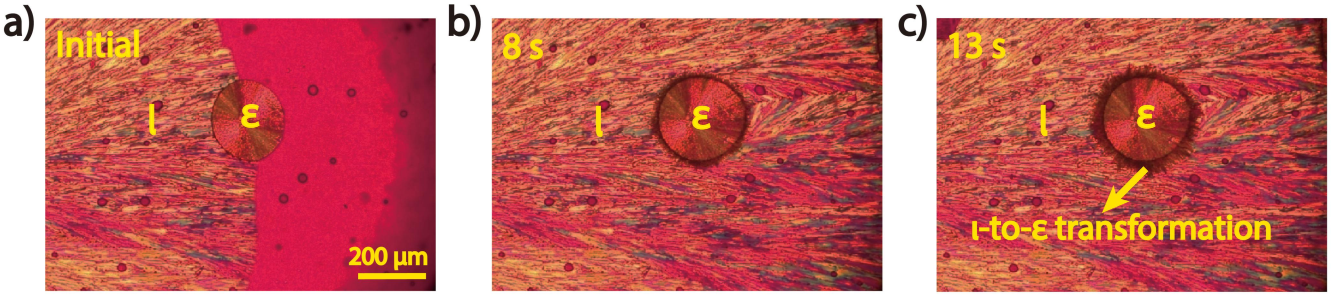

Supplementary Figure 19. Polymorphic conversion from Form ι to Form ε. (a) Initial; (b) 8s; (c) 13 s

9. Supplementary Methods

The methods for preparing crystallographically pure samples of the NIC polymorphs are described below.

Form α. The commercially available sample of NIC was Form α.

Form β. To prepare a crystallographically pure Form β, Form θ was first prepared at 60-70 °C, possibly concomitant with Forms δ and ζ. After Form θ partially converted to Form β, the sample was heated to a temperature just below the melting point of Form β to melt Form θ and other possibly present metastable polymorphs, followed by cooling to 100 °C to grow pure Form β.

Form γ. To prepare a crystallographically pure Form γ sample, an NIC melt was quenched at 30-80 °C to nucleate Form δ. After Form δ partially converted to Form γ, this sample was heated just below the melting point of Form γ to melt Form δ and other possible metastable polymorphs. Then, the seeds of Form γ were grown at 105 °C, yielding a pure Form γ sample.

Form δ. A crystallographically pure Form δ sample can be obtained by crystallizing an NIC melt at room temperature. However, this Form δ sample rapidly converted to Form γ.

Form ε. Crystallization of an NIC melt at 70-80 °C could yield a crystallographically pure Form ε sample or a Form ε sample concomitant with Forms δ and θ.

Form ζ. Form ζ usually cross-nucleates on the surface of Form γ between 90-95 °C or Form δ between 90-105 °C. A crystallographically pure ζ-NIC sample could be obtained by seeding ζ-NIC seeds in a fresh melt droplet at 105 °C.

Form η. Cold crystallization at 104 °C randomly yielded pure Form η or a concomitant sample containing other polymorphs. The nucleation probability of Form η was found to be extremely low.

Form θ. A crystallographically pure Form θ sample can be obtained by nucleating an NIC melt at 60 °C and consuming all materials without polymorphic conversion to Form β. Form θ often concomitantly nucleates with Forms δ and ζ and then converts to Form β. Therefore, obtaining pure θ is a probabilistic event.

Form ι. Seeding iso-nicotinamide (iso-NIC) Form I at the edge of an NIC melt sample between 70-95 °C randomly triggered the nucleation of Form α and/or Form ι. Because Form α always nucleates from the site of seeding and converts Form ι, it is difficult to obtain pure Form ι.
