# Supplementary material for: Rich polymorphism in nicotinamide revealed by melt crystallization and crystal structure prediction
Source: Commun Chem. 2020 Nov 4;3:152. doi: 10.1038/s42004-020-00401-1 (PMC9814109; doi:10.1038/s42004-020-00401-1)
Supplement: Supplementary file 2 — Description of Additional Supplementary Files [file 42004_2020_401_MOESM2_ESM.pdf]

Description of Additional Supplementary Files

File Name: Supplementary Data 1

Description: A integrated CIF file, containing nine single-crystal structures of nicotinamide polymorphs.

File Name: Supplementary Data 2

Description: A integrated CIF file, containing 124 structures of nicotinamide predicted by crystal structure prediction.

File Name: Supplementary Movie 1

Description: A video of the single-crystal cultivation of nicotinamide Form γ from the melt microdroplet.
